# AAV-Mediated CAG-Targeting Selectively Reduces Polyglutamine-Expanded Protein and Attenuates Disease Phenotypes in a Spinocerebellar Ataxia Mouse Model

**DOI:** 10.3390/ijms25084354

**Published:** 2024-04-15

**Authors:** Anna Niewiadomska-Cimicka, Lorraine Fievet, Magdalena Surdyka, Ewelina Jesion, Céline Keime, Elisabeth Singer, Aurélie Eisenmann, Zaneta Kalinowska-Poska, Hoa Huu Phuc Nguyen, Agnieszka Fiszer, Maciej Figiel, Yvon Trottier

**Affiliations:** 1 Institute of Genetics and Molecular and Cellular Biology, INSERM U1258, CNRS UMR7104, University of Strasbourg, 67404 Illkirch, France; lorraine.fievet@hotmail.fr (L.F.); keime@igbmc.fr (C.K.); virly@igbmc.fr (A.E.); 2 Department of Molecular Neurobiology, Institute of Bioorganic Chemistry, Polish Academy of Sciences, 61-704 Poznan, Poland; msurdyka@ibch.poznan.pl (M.S.); jesion.ewelina@gmail.com (E.J.); zkalinowska@ibch.poznan.pl (Z.K.-P.); mfigiel@ibch.poznan.pl (M.F.); 3 Centre for Rare Diseases (ZSE), University of Tuebingen, 72076 Tuebingen, Germany; elisabeth.singer@med.uni-tuebingen.de; 4 Institute of Medical Genetics and Applied Genomics, University of Tuebingen, 72076 Tuebingen, Germany; 5 Department of Human Genetics, Medical Faculty, Ruhr University Bochum, 44801 Bochum, Germany; huu.nguyen-r7w@ruhr-uni-bochum.de; 6 Department of Medical Biotechnology, Institute of Bioorganic Chemistry, Polish Academy of Sciences, Noskowskiego 12/14, 61-704 Poznan, Poland; agnieszka.fiszer@ibch.poznan.pl

**Keywords:** CAG-targeting therapy, allele selectivity, short hairpin RNA, adeno-associated virus, CAG repeat expansion, *Spinocerebellar ataxia*

## Abstract

Polyglutamine (polyQ)-encoding CAG repeat expansions represent a common disease-causing mutation responsible for several dominant spinocerebellar ataxias (SCAs). PolyQ-expanded SCA proteins are toxic for cerebellar neurons, with Purkinje cells (PCs) being the most vulnerable. RNA interference (RNAi) reagents targeting transcripts with expanded CAG reduce the level of various mutant SCA proteins in an allele-selective manner in vitro and represent promising universal tools for treating multiple CAG/polyQ SCAs. However, it remains unclear whether the therapeutic targeting of CAG expansion can be achieved in vivo and if it can ameliorate cerebellar functions. Here, using a mouse model of SCA7 expressing a mutant Atxn7 allele with 140 CAGs, we examined the efficacy of short hairpin RNAs (shRNAs) targeting CAG repeats expressed from PHP.eB adeno-associated virus vectors (AAVs), which were introduced into the brain via intravascular injection. We demonstrated that shRNAs carrying various mismatches with the CAG target sequence reduced the level of polyQ-expanded ATXN7 in the cerebellum, albeit with varying degrees of allele selectivity and safety profile. An shRNA named A4 potently reduced the level of polyQ-expanded ATXN7, with no effect on normal ATXN7 levels and no adverse side effects. Furthermore, A4 shRNA treatment improved a range of motor and behavioral parameters 23 weeks after AAV injection and attenuated the disease burden of PCs by preventing the downregulation of several PC-type-specific genes. Our results show the feasibility of the selective targeting of CAG expansion in the cerebellum using a blood–brain barrier-permeable vector to attenuate the disease phenotype in an SCA mouse model. Our study represents a significant advancement in developing CAG-targeting strategies as a potential therapy for SCA7 and possibly other CAG/polyQ SCAs.

## 1. Introduction

Polyglutamine (PolyQ) spinocerebellar ataxias (SCA), including SCA1, SCA2, SCA3, SCA6, SCA7, and SCA17, are diseases caused by the expansion of CAG trinucleotide repeats located in the coding region of specific genes, which results in an abnormally elongated polyQ tract in the corresponding proteins [1]. PolyQ diseases also include Huntington’s disease (HD) and dentatorubral-pallidoluysian atrophy (DRPLA), which are all dominantly inherited. The presence of 33–39 or more CAGs in the tract in only one of the gene alleles is regarded as pathological. The longer the expanded CAG repeats, the earlier and more severe the disease symptoms are. Moreover, the CAG repeat tract is unstable and has the propensity to further expand in somatic tissues, accelerating the disease progression [2]. The motor symptoms in SCA arise primarily from progressive degeneration of the cerebellum, brainstem, and associated structures [3]. In the cerebellum, Purkinje cells (PCs) are the neuronal population most frequently affected [4]. There is still no effective therapy to prevent or significantly delay the neurodegenerative process of CAG/polyQ SCAs.

WT SCA proteins are widely expressed and essential for various cellular functions [5]. The polyQ expansion causes a conformational change and misfolding of proteins, which become toxic, triggering a complex cascade of cellular dysfunctions leading to neurodegeneration [4,5]. Therefore, the direct therapeutic strategy is based on lowering the disease-causing RNA and/or the protein level to prevent the pathogenic pathway at early stages. To this end, over several years, various therapeutic approaches have been developed, making use of DNA-based antisense oligonucleotides (AON), RNA interference (RNAi) molecules, or DNA editing techniques [5,6,7].

Lowering gene expression can either be allele-selective only towards the mutant gene or non-selective [6]. However, the non-selective strategy, in which both mutant and WT alleles are targeted, may represent a risk factor for patients, especially when considering that such a treatment might last for decades. Allele-selective lowering strategies based on single nucleotide polymorphism (SNP) variants linked to CAG-expanded alleles can discriminate mutant over WT transcripts. However, this approach has its limitations, as suitable SNPs are rare in the population, and the development of several molecules would be necessary to benefit a substantial number of patients. An alternative is to target expanded CAG tracts directly and selectively with a single reagent, which could be suitable for all CAG/polyQ diseases [8,9]. For this purpose, synthetic CUG-based RNAi molecules were designed that contain nucleotides introducing single or multiple base mismatches with the target CAG-expanded sequence. The “mismatch” concept is used to weaken or prevent AGO2 cleavage after the binding of such a reagent with the CAG tract in the transcript. This results in the activation of a microRNA-like mechanism that is preferential for mutant alleles in which multiple oligonucleotides can bind, ensuring cooperative and efficient silencing [10,11].

Previous work showed that self-duplexing, guide-strand-only small interfering RNAs (sd-siRNA) carrying various mismatches with the target CAG-expanded sequence were highly efficient in selectively lowering the level of polyQ-expanded Huntingtin (HTT) in fibroblasts of HD patients [8]. In particular, one of these sd-siRNAs, a 7 GCU-repeats containing a single U> A substitution at position 9, named A2, also lowered the level of mutant ATXN3, ATXN7, and ATN1 involved in SCA3, SCA7, and DRPLA, respectively, and represents a potential universal reagent for CAG/polyQ disorders [10,12,13]. Furthermore, putting the sequence of A2 into the 3′ arm of the shRNA stem (named A2R) also led to allele-selective lowering of these mutant proteins in patient fibroblasts [8,14]. This result demonstrates the feasibility of converting active sd-siRNA reagents into vector-expressed shRNAs to achieve a long-term lowering effect. To date, only a few studies have provided evidence of the efficacy of a CAG repeat-targeting strategy in preclinical animal models [9,15,16,17].

The aim of the current study was to develop and validate a therapeutic approach applicable to CAG/polyQ SCAs using a CAG repeat-targeting strategy to lower the expression of expanded-polyQ proteins in vulnerable cerebellar neurons. To this end, we tested various CAG repeat-targeting shRNAs, including A2R, expressed using the PHP.eB adeno-associated virus serotype (AAV PHP.eB), which can widely transduce brain cells after intravenous systemic administration [18]. AAV vectors expressing shRNAs were administrated to the SCA7^140Q/5Q^ knock-in mouse model, which expressed the WT mouse allele with 5 CAGs and a mutant Atxn7 allele with an expansion of 140 CAGs. These allele sizes fall within the range of normal ATXN7 alleles in the human population, which mostly vary between 4 and 18 CAGs [19], and mutant alleles causing the early onset of SCA7, which are typically >70 and up to 460 CAGs [20,21,22]. SCA7^140Q/5Q^ mice recapitulate several phenotypes observed in early onset patients, including the progressive impairment of motor performance, which coincides with morphological and electrophysiological alterations of PCs and the downregulation of PC-type-specific genes [23]. Using the AAV-based approach, we show that the different CAG-repeat targeting shRNAs have different efficacy in lowering the ATXN7 level in the cerebellum and various safety profiles. We reported that shRNA A4 leads to the selective lowering of mutant ATXN7 (mATXN7) in the SCA7 mouse cerebellum, without affecting the level of normal ATXN7 (nATXN7), and improves motor, behavioral, and molecular phenotypes 23 weeks after systemic injection. Thus, our results support the therapeutic potential of the CAG repeat-targeting strategy for the treatment of CAG/polyQ SCA diseases.

## 2. Results

### 2.1. A2R shRNA Expressed from AAV PHP.eB Lowers ATXN7 in SCA7 Mouse Primary Cells

**Figure 1:**
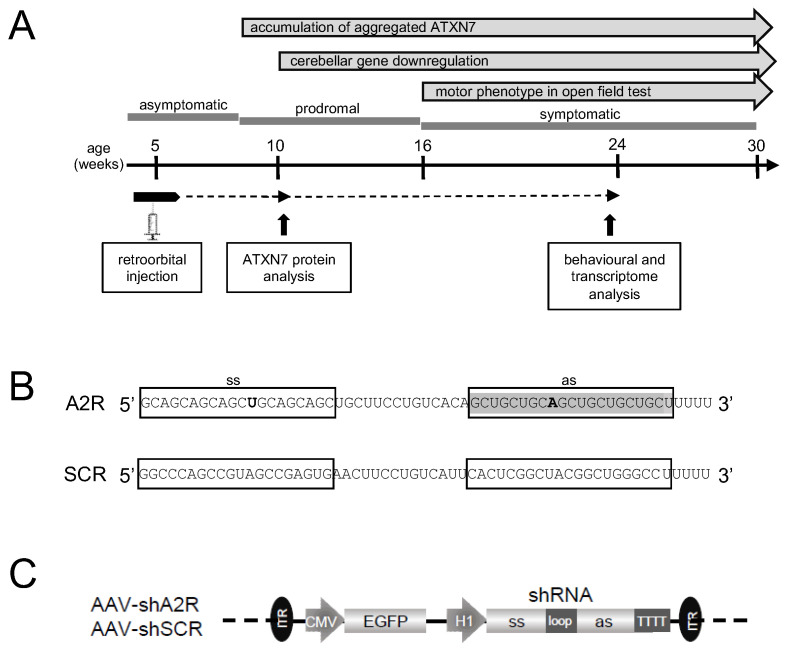
Experimental design: (**A**) Timeline of disease onset and progression in SCA7^140Q/5Q^ knockin mice. Mice were injected retro-orbitally into their blood stream at an asymptomatic stage of 5–6 weeks of age. A prodromal period is characterized by progressive molecular alterations, notably the accumulation of aggregated mATXN7 observed at 8–10 weeks and the downregulation of PC type-specific genes from 12 weeks. The symptomatic period is characterized by progressive motor and behavioral dysfunctions that manifest from 16 weeks, as revealed by an open field test. The lifespan of SCA7 mice is shortened to about 55–60 weeks. (**B**) Nucleotide sequence of A2R and scrambled (SCR) shRNAs. Ss, sense strand; as, antisense strand. The as of shA2R corresponds to A2 sd-siRNA [8]. (**C**) Construct of AAV PHP.eB vector containing two expression cassettes: CMV promoter driving eGFP to trace the efficiency of AAV transduction in vivo and H1 promoter driving shA2R or shSCR. ITR, inverted terminal repeats.

Since A2R shRNA (shA2R) has already been shown to efficiently lower the level of mATXN7 in SCA7 patient fibroblasts [13,14], we first aimed to assess the lowering effect of A2R shRNA expressed from the AAV PHP.eB vector intravascularly injected in asymptomatic SCA7^140Q/5Q^ mice (Figure 1A). As a first step towards in vivo evaluation, we first applied an ex vivo approach to test the efficacy of shA2R in the mouse context. We transduced SCA7^140Q/5Q^ mouse primary cells with an AAV PHP.eB vector either expressing shA2R or an shRNA with scrambled A2R sequences (shSCR) (Figure 1B,C). The synthetic sd-siRNAs A2 and A2M (a potent chemically modified A2 [12]) were also transfected into these cells for analysis. Protein extracts of transduced or transfected cells were analyzed by western blot using specific anti-ATXN7 antibody. Protein extracts from untreated WT and SCA7^140Q/140Q^ homozygous mice were used as controls to accurately distinguish the nATXN7 from mATXN7 migration on blots. When compared to shSCR, we observed that shA2R selectively decreased the level of mATXN7 in a dose-dependent manner in mouse primary cells (Figure S1A,B). Similarly, compared to the control siRNA, A2 and A2M lowered the level of mATXN7 (Figure S1C,D). The results confirmed that shRNA A2R and synthetic A2 reagents have a lowering effect on the mouse mATXN7 protein but not on mouse nATXN7.

### 2.2. Dose-Dependent Transduction of the Cerebellum and Safety Profile of AAV PHP.eB Injected Systemically

**Figure 2:**
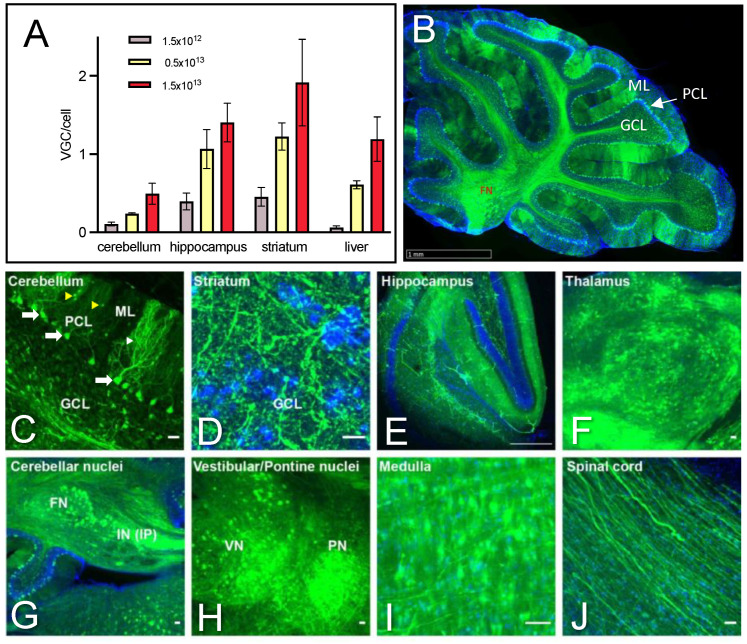
AAV PHP.eB efficiently transduces cerebellar and cerebellar-associated structures. (**A**) Viral genome copy per cell (vgc/cell) in different mouse tissues 4 days after injection of AAV PHP.eB at increasing doses (vg/kg). Data are expressed as mean ± SEM (*n* = 4/tissue). (**B**) Detection of eGFP signal (green) in the majority of PCs on sagittal sections of the cerebellum of mice injected with 1.5 × 10^13^ vg/kg and analyzed 4 weeks post-injection. (**C**) Higher magnification of PC showing eGFP-positive soma (white arrows) and dendritic arbors (white arrowhead). A number of cells in the molecular layer (yellow arrowheads) (likely stellate and basket neurons) were also eGFP-positive. (**D**–**E**) An eGFP-positive signal was observed in fibers of striatal neurons (**D**) and the hippocampal molecular layer (**E**). (**F**–**J**) An eGFP-positive cell soma was detected at high density in the thalamus (**F**), cerebellar nuclei (**G**), vestibular and pontine nuclei (**H**), and the medulla (**I**), and the spinal cord shows eGFP-positive fibers (**J**). FN, fastigial nucleus; GCL, granule cell layer; IP, interposed nucleus; IN, intracerebellar nucleus; ML, molecular layer; PC, Purkinje cell; PCL, PC layer. Scale bars: ((**B**) 1 mm; (**C**,**J**) 10 µm; (**D**) 5 µm; (**E**–**I**) 50 µm).

The AAV PHP.eB capsid can cross the blood–brain barrier and transduce brain cells following systemic injection in mouse strains expressing the Ly6a capsid receptor, such as the C57BL/6J background of SCA7^140Q/5Q^ mice. To characterize the transduction efficiency of AAV PHP.eB expression vectors in the cerebellum, we performed retro-orbital vascular injections at different doses (1.5 × 10^12^, 0.5 × 10^13^, and 1.5 × 10^13^ virus genomes per kg (vg/kg)) into 4.5–5.5 weeks old mice. Figure 2A shows a dose-dependent increase of vg copies per cell (vgc/cell) in the cerebellum, as well as the striatum, hippocampus, and liver. As reported previously [24], the striatum and hippocampus showed higher transduction efficiency than the cerebellum and liver. At the lowest dose (1.5 × 10^12^ vg/kg), viral transduction was barely detected in the cerebellum, while at the highest dose (1.5 × 10^13^ vg/kg), the transduction efficiency ranged from 0.5 vgc/cell in the cerebellum to 2 vgc/cell in the striatum.

As our AAV PHP.eB vectors expressed an eGFP reporter cassette (Figure 1C), we confirmed the broad distribution of eGFP fluorescence in the cerebellum on sagittal brain sections of mice injected with 1.5 × 10^13^ vg/kg (Figure 2B). Importantly, the majority of PCs in the cerebellum were efficiently transduced by AAV PHP.eB. A number of cells in the molecular layer were also eGFP-positive, while granule neurons in the granule cell layer of the cerebellar cortex appeared not or only scarcely transduced (Figure 2C). Other well-transduced regions based on eGFP signal included the striatum and hippocampus, as well as the thalamus (Figure 2D–F). Interestingly, the cerebellar nuclei and cerebellar-associated structures, such as the vestibular/pontine nuclei and medulla, had a high density of eGFP-positive cell soma, and the spinal cord had eGFP-positive fibers (Figure 2G–J). Together, the results confirmed that the retroorbital injection of AAV PHP.eB widely targets PCs and transduces several brain structures involved in motor coordination.

To ensure that the injection of AAV has no confounding effect per se, SCA7 mice were injected with AAV-shSCR or saline. Brain tissues were analyzed 4 weeks later for the expression levels of nATXN7 and mATXN7 and for the possible activation of an immune response. Western blot showed that the injection of up to 3.0 × 10^13^ vg/kg of AAV-shRNA had no effect on the expression levels of nATXN7 and mATXN7 (Figure S2A,B) or on immune system markers, such as Interferon B (INFB) and Interferon Regulatory Factor 7 (IRF7), when compared to the saline treatment (Figure S2C,D). Moreover, mice injected at this dose did not show abnormal behavior.

### 2.3. AAV-shA2R Lowers mATXN7 Cerebellar Protein Level and Induces Severe Adverse Side Effects

**Figure 3:**
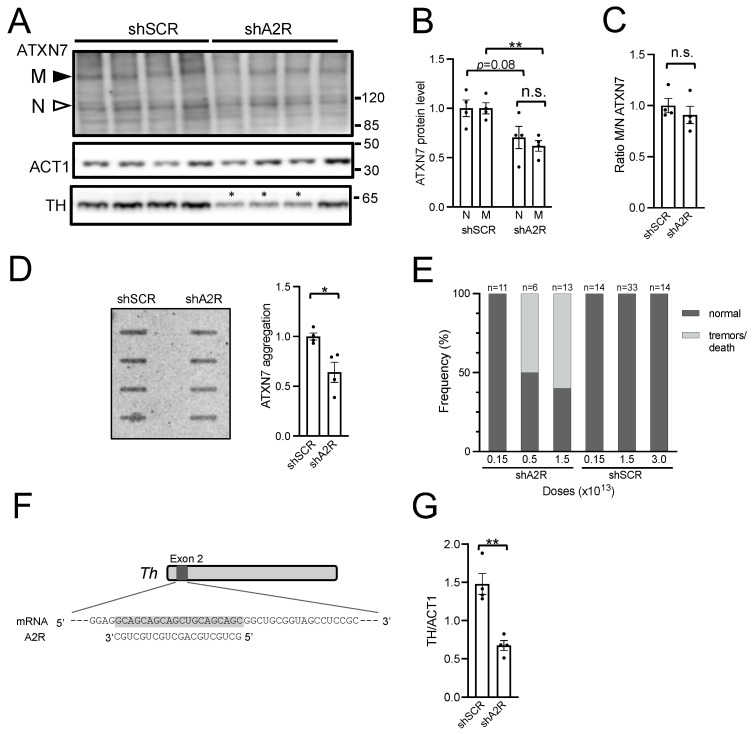
Analysis of the efficacy, allele selectivity, and safety of AAV-shA2R injection in SCA7 mice. (**A**) Representative western blot analysis of soluble normal (N) and mutant (M) ATXN7, Actin-1 (ACT1), and Tyrosine hydroxylase (TH) in the cerebellum of SCA7 mice injected with AAV-shA2R or AAV-shSCR at 1.5 × 10^13^ vg/kg. Mice were injected at the age of 6 weeks and were sacrificed at 10 weeks for analysis. The asterisk indicates 3 mice that developed a tremor-like phenotype. (**B**) Signal intensities of soluble nATXN7 and mATXN7 were normalized to ACT1 levels and plotted relative to shSCR conditions with the mean set at 1. (**C**) Ratio of signal intensities of mATXN7/nATXN7 normalized to ACT1 levels. (**D**) Signal intensities of aggregated mutant ATXN7 in cerebellar protein extracts assessed using on filter trap assay. The intensities were plotted relative to SCR conditions. (**E**) Dose dependent adverse side effects of shA2R. The affected mice (light grey) either presented with a tremor phenotype and had to be sacrificed or died during the course of the experiment. (**F**) A 20-nucleotide complementarity to the antisense sequence of shA2R with the mouse Th transcript. (**G**) Signal intensities of TH normalized to ACT1 levels. Data are expressed as mean± SEM and were analyzed using two-tailed Student’s *t*-test. * *p* < 0.05; ** *p* < 0.01. n.s. for not significant.

To assess shA2R activity in vivo, we performed retroorbital injections of AAV-shA2R or AAV-shSCR at 1.5 × 10^13^ vg/kg in asymptomatic 6-week-old SCA7 mice. The cerebellum and other brain regions were analyzed at 10 weeks of age for their SDS-soluble ATXN7 protein content. The expression of shA2R in the cerebellum significantly decreased mATXN7 level by 38% (*p* = 0.003) compared to the level in shSCR samples and also decreased nATXN7 by 29%, however, less statistically significant (*p* = 0.08) (Figure 3A,B). Despite the reduction of soluble protein levels, the ratio of mATXN7/nATXN7 in shA2R samples was comparable to that in shSCR samples, underlining the absence of allele selectivity (Figure 3C). We previously showed that part of mATXN7 aberrantly accumulates as insoluble oligomers in the cerebellum of SCA7 mice from 8 weeks of age [23]. A filter trap assay showed that the aggregated forms of mATXN7 were reduced by 36% (*p* = 0.016) in the cerebellum of AAV-shAR2-treated mice (Figure 3D), consistent with the reduced level of soluble mATXN7 quantified on the western blot. In contrast, the analysis of hippocampal and striatal samples showed no decrease of soluble mATXN7 by shA2R when compared to shSCR (Figure S3).

We noted that the systemic injection of AAV-shA2R led to substantial adverse side effects affecting both WT and SCA7 mice in a dose-dependent manner. About 50–60% of mice injected with ≥0.5 × 10^13^ vg/kg AAV-shA2R developed tremors or died, in some cases as early as 2 weeks post-injection (Figure 3E). Necropsy analysis revealed no obvious anomalies of internal organs such as the heart, liver, kidney, and gastrointestinal tract, which would explain mortality. Animals injected with 1.5 × 10^12^ vg/kg of AAV-shA2R did not show adverse phenotypes at the terminal point of the study (27 weeks post-injection). However, at this low dose, shA2R did not lower mATXN7 significantly. In contrast to AAV-shA2R, the injection of AAV-shSCR at doses up to 3.0 × 10^13^ vg/kg was harmless, suggesting that the toxicity of AAV-shA2R may be related to off-targets. With this in mind, bioinformatic analysis revealed seven potential off-target transcripts (100% complementarity to ≥20 nt) of shA2R-processed sequences in Mus musculus (Table S1). For these transcripts, it could be assumed that RNA released from shA2R acted as siRNA, inducing endonucleolytic cleavage and degradation, even if a single binding site was present in the transcript. One of these transcripts, the Tyrosine hydroxylase (Th), is a potential off-target in mice (Figure 3F) but not in humans. Th has a high expression level in specific brain regions, including the cerebellum, where expression is specific to PCs (Figure S4A). Mutations leading to the reduction of TH activity or levels in mice cause neurological symptoms, including tremors [25,26]. Western blot analysis showed that the TH level was decreased on average by 55% (*p* = 0.018) in the cerebellum of AAV-shA2R-treated mice (Figure 3A,G), while AAV-shSCR-treated mice showed no difference compared to saline-treated mice (Figure S4B,C). Interestingly, out of four AAV-shA2R-treated mice, three showed a strong decrease in TH level in the cerebellum and tremors (asterisks in Figure 3A), while the fourth mouse had a milder TH decrease and no tremors. In the striatum and hippocampus, TH expression levels were similar in AAV-shA2R- and AAV-shSCR-treated mice (Figure S3E). This suggested that decreasing the TH level below a certain threshold in the cerebellum may account for the adverse phenotype.

The results demonstrated that shA2R has the potential to decrease the level of soluble and aggregated forms of mATXN7 in the mouse cerebellum. However, shA2R causes adverse side effects such as abnormal behaviors, lethality, and off-targeting of Th, precluding further investigation of its therapeutic potential in mice.

### 2.4. AAV PHP.eB Expressing A4 shRNA Selectively Lowers mATXN7 Cerebellar Level and Is Well Tolerated

**Figure 4:**
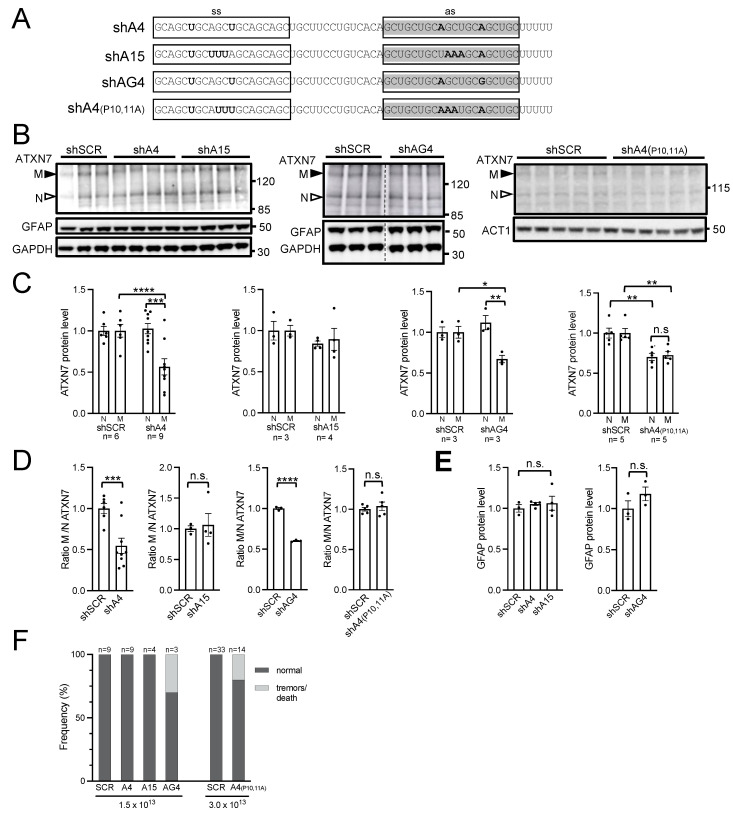
In vivo analysis of the efficacy, allele selectivity, and safety of shRNAs A4, A15, AG4, and A4(P10,11). (**A**) Nucleotide composition of shRNAs. ss, sense strand; as, antisense strand. The as of shA4 and shA15 corresponds to A4 sd-siRNA and A15 sd-siRNA [8]. (**B**) Representative western blot analysis of ATXN7, ACT1, GFAP, and GAPDH in the cerebellum of SCA7 mice injected with different AAV vectors. The 5-week-old SCA7 mice were injected at 1.5 × 10^13^ vg/kg with AAV-shA4, AAV-shA15, AAV-shAG4, or AAV-shSCR, and at 3.0 × 10^13^ vg/kg with AAV-shA4(P10,11A) and AAV-shSCR. Mice were sacrificed at 5 to 7 weeks post-injection for analysis, depending on the experimental group. Note that the blot in the middle panel was spliced in the center (vertical dotted line) to remove samples from an unrelated treatment and did not have other manipulation. (**C**) Signal intensities of normal (N) and mutant (M) ATXN7 normalized to GAPDH or ACT1 levels and plotted relative to SCR conditions with mean set at 1. The graph for the experimental group AAV-shA4 and AAV-shSCR combined data from two independently injected cohorts of mice. (**D**) Ratio of signal intensities of mATXN7/nATXN7 normalized to GAPDH or ACT1 levels and plotted relative to SCR conditions with the mean set at 1. (**E**) Signal intensities of GFAP normalized to GAPDH levels and plotted relative to SCR conditions with mean set at 1. (**F**) Frequency of adverse side effects of the different AAV-shRNAs injected at the indicated doses. Data are expressed as mean± SEM and were analyzed using two-tailed Student’s *t*-test. * *p* < 0.05; ** *p* < 0.01; *** *p* < 0.001; **** *p* < 0.0001. n.s. for not significant.

To improve the safety profile of shRNA, we generated a series of new molecules with various nucleotide substitutions that mismatched the targeted CAG repeats and that reduced the off-targeting potential. Previous studies identified two siRNA reagents, named A4 and A15, which showed allele-selective efficacy to lower mutant HTT in HD patient fibroblasts, similarly to the A2 reagents [8]. A4 and A15 sequences can be distinguished from A2 by the presence of different nucleotide substitutions (Figure 4A). A4 siRNA has a lower number of potential off-targets than A2 siRNA in mice (two versus six primary off-targets), while A15 has no such off-target (Table S1). Until now, A4 and A15 siRNA sequences have not been tested in the context of an shRNA expression cassette, nor for their efficacy, selectivity, and safety in in vivo models of polyQ diseases. Therefore, we introduced them in AAV PHP.eB to generate AAV-shA4 and AAV-shA15, and we proceeded to perform systemic injections at a dose of 1.5 × 10^13^ vg/kg in SCA7 mice, as previously conducted for shA2R (Figure 4A). In addition, we tested two shRNAs derived from A4 (named shAG4 and shA4 (P10,11A)) containing additional nucleotide substitutions to decrease the number of off-targets (Figure 4A; Table S1).

The western blot analysis of cerebellar protein extracts revealed that shA4 and shAG4 selectively decreased the mATXN7 level by 50% (*p* = 0.0009) and 32% (*p* = 0.018), respectively, compared to the control shSCR, while shA15 had no effect on ATXN7 levels (Figure 4B,C). Moreover, the ratio of mATXN7/nATXN7 in shA4 and shAG4 samples was also significantly reduced by 53% (*p* = 0.0005) and by 40% (*p* = 0.00012), respectively (Figure 4D). The injection of 3.0 × 10^13^ vg/kg, AAV-shA4(P10,11A) decreased ATXN7 in the cerebellum but performed with a similar effect for mATXN7 (by 27%, *p* = 0.0046) and nATXN7 (by 30%, *p* = 0.0041). Hence, shA4(P10,11A) did not modify the ratio of mATXN7/nATXN7 (Figure 4B–D), which demonstrates the absence of allele selectivity.

In the striatum, shA4 also showed a tendency to selectively decrease mATXN7 by 56%; however, it was less statistically significant (*p* = 0.08), resulting in a reduced mATXN7/nATXN7 ratio by 50% (*p* = 0.049) (Figure S5A–C). In the hippocampus, shA4 decreased mATXN7 by 39% (*p* = 0.005) but also reduced the level of nATXN7 slightly and, hence, did not significantly modify the ratio of mATXN7/nATXN7. shAG4 lowered both nATXN7 and mATXN7 by 49% (*p* = 0.011 and *p* = 0.004, respectively) in the hippocampus and showed no lowering effect in the striatum (Figure S5A–C).

None of these shRNAs induced the gliosis marker glial fibrillary acidic protein (GFAP) in the different brain tissues (Figure 4B; Figures S4B,D and S5A,D). AAV-shA4 and AAV-shA15, as well as AAV-shSCR, did not induce any deleterious phenotype, whereas several mice injected with AAV-shAG4 or AAV-shA4(P10,11A) died during the course of the experiments (Figure 4F). Considering that AAV-shA4 selectively reduced the mATXN7 level in the SCA7 mouse cerebellum and striatum and was well tolerated, we further analyzed its safety by testing whether AAV-shA4 may have a general effect on the transcriptome. To this end, we performed an RNA-seq analysis of the cerebella of WT mice treated with AAV-shA4 (1.5 × 10^13^ vg/kg, at 9 weeks post-injection) and untreated. From 16,100 protein-coding genes, only 36 genes were deregulated with a fold change (FC) > 1.3 (adjusted *p* < 0.05) between AAV-shA4-treated WT and untreated WT mice and none with FC > 1.7 (Figure S6; Table S2). Noteworthy, none of the 36 downregulated genes was a potential A4 off-target as defined in silico or contained CAG repeats in the mRNA sequence. Gene ontology (GO) analysis of the 36 differentially expressed genes (DEGs) revealed no GO term enrichment. Therefore, the results indicated that the effect of shA4 on the cerebellar transcriptome was modest in WT mice.

### 2.5. AAV-shA4 Improves Motor, Behavioral, and Molecular Phenotypes in SCA7 Mice

**Figure 5:**
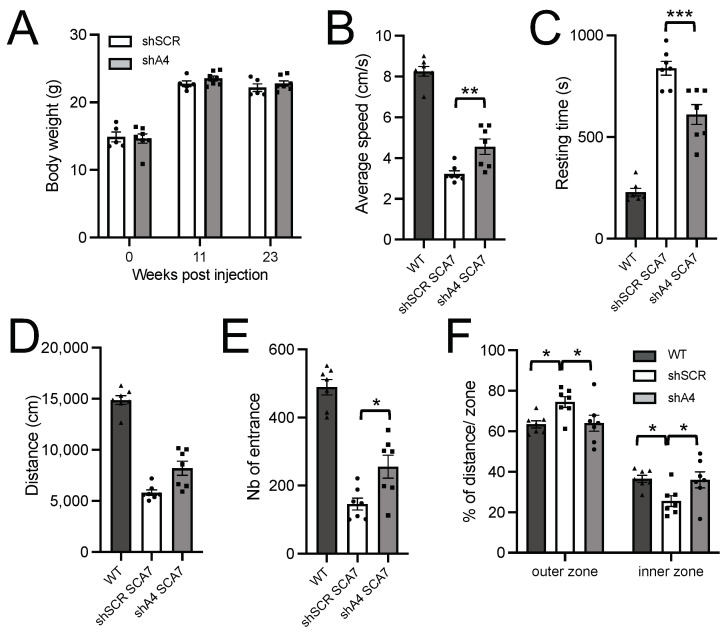
Improvement of sensorimotor activity of SCA7 mice treated with AAV-shA4. (**A**) Post-injection body weight of SCA7 mice treated with AAV-shSCR or AAV-shA4 at 5 weeks of age. Data are mean ± SEM (n = 7 SCA7 males for shA4 and 5 SCA7 males for shSCR). Ordinary two-way ANOVA. (**B**–**F**) Open field analyses of SCA7 mice treated with AAV-shA4 and AAV-shSCR, and age-matched WT mice. AAV-shA4-treated SCA7 mice had improved locomotor performance and exploration activity at 23 weeks post-injection, compared to control AAV-shSCR-treated SCA7 mice. Data are mean ± SEM. One-way ANOVA followed by post hoc Tukey’s test. * *p* < 0.05; ** *p* < 0.01; *** *p* < 0.001.

SCA7 adult mice were shown to plateau at a lighter weight than WT mice and to show deficits in various sensori-motor tests [23]. On the basis of the preceding phenotypic data, we estimated that a therapeutic treatment leading to a 25% improvement in body weight and open field test performance was likely to be significantly detected from 16 weeks of age at *p* < 0.05 with a power greater than 80%. To assess whether the lowering effect of AAV-shA4 provides a beneficial effect on the phenotype, we proceeded with intravascular injections of 5-week-old SCA7 mice. AAV-shA4 treatment did not improve the body weight of mice tested at 11 and 23 weeks post-injection when compared to the control AAV-shSCR (Figure 5A). However, at 23 weeks post-injection, SCA7 mice treated with AAV-shA4 showed improved spontaneous locomotor activity. In open field tests of 30 min duration, treated mice had an increased average speed (by 26%, *p* = 0.0078), a reduced resting time (by 37%, *p* = 0.0008), and, hence, traveled longer distances (by 26%, *p* = 0.0082) than control mice (Figure 5B–D). Moreover, AAV-shA4-treated SCA7 mice entered more frequently in the inner zone of the open field than AAV-shSCR-treated mice (Figure 5E), and they had a ratio of inner/outer zone traveling distance similar to WT mice (Figure 5F), suggesting a reduction in thigmotaxis.

**Figure 6:**
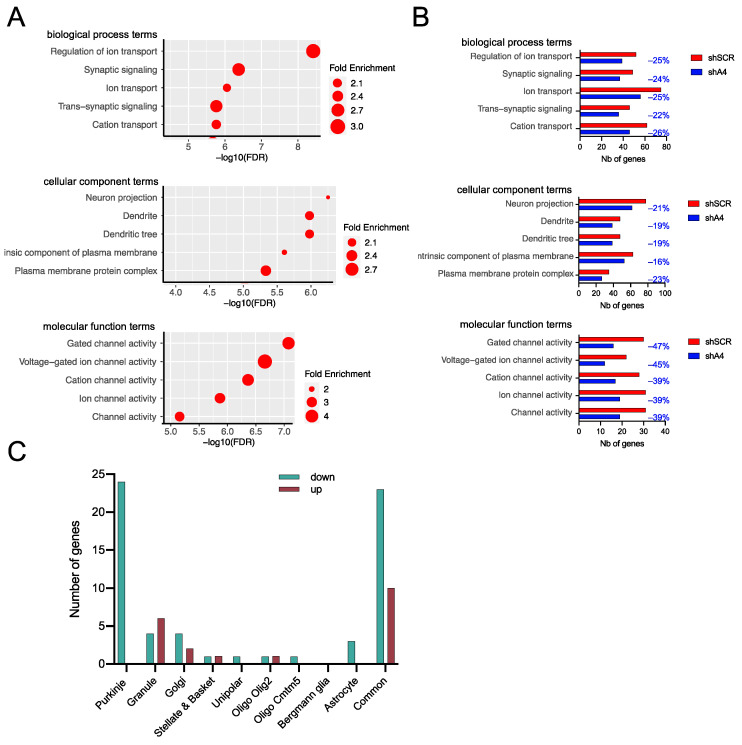
Analysis of differentially expressed genes in AAV-shSCR- and AAV-shA4-treated SCA7 mice compared to WT mice. (**A**) Gene ontology (GO) term enrichment for the 426 DEGs between AAV-shSCR-treated SCA7 mice (*n*= 5 males) and WT mice (*n* = 4 males) calculated with ShinyGO v0.80. The 5 most enriched GO terms of each category are sorted by –Log10(FDR). (**B**) Comparison of the number of DEGs accounting for each major enriched GO term in the transcriptomes of AAV-shSCR- and AAV-shA4-treated SCA7 mice, relative to WT mice. The values in blue represent the percentages of genes (–%) that are deregulated in AAV-shSCR but are restored to a FC < 1.3 or are not deregulated in AAV-shA4-treated SCA7 mice anymore. (**C**) Cell type-specific distribution of genes that are deregulated in AAV-shSCR but are restored to an FC < 1.3 or are not deregulated in AAV-shA4-treated SCA7 mice anymore. Among the 140 genes deregulated only in AAV-shSCR-treated SCA7 mice, 82 genes were assigned to one or more cerebellar cell types.

Development of the sensorimotor phenotypes in SCA7 mice correlated with the deregulation of cerebellar genes, including the downregulation of PC type-specific genes [23]. To assess whether AAV-shA4 treatment ameliorates the expression of deregulated genes, we performed RNA-seq analysis of the cerebellum of AAV-shA4-treated SCA7 mice at 23 weeks post-injection and compared them to AAV-shSCR-treated SCA7 mice and age-matched untreated WT mice. Compared to WT mice, AAV-shSCR-treated SCA7 mice showed 426 DEGs with an FC > 1.3 (adjusted *p* < 0.05), including 311 down-regulated and 115 up-regulated genes (Figure S7A). GO analysis revealed high enrichment in terms associated with ion transport and synaptic signaling biological processes, dendritic cellular components, and ion channel molecular functions (Figure 6A). These enrichments are consistent with enriched GO terms reported previously for DEGs in the cerebellum of 40-week-old untreated SCA7 mice [23] (Figure S8) and could constitute a signature of SCA7 disease progression in mice. Using available datasets [23], we assigned cerebellar cell-type-specific distribution to 269 of 426 DEGs, and we subsequently determined that 104 PC type-specific genes were downregulated in AAV-shSCR-treated SCA7 mice (Figure S7B). This confirms that PC pathology represents a major characteristic of AAV-shSCR-treated SCA7 mice, as previously reported for untreated SCA7 mice [23], and this shows that the AAV transduction per se does not affect the pathology in these neurons.

We then looked at the status of these 426 genes in AAV-shA4-treated SCA7 mice. A total of 286 genes were still significantly deregulated with FC > 1.3 when compared to WT mice. Interestingly, 140 genes were restored to a FC < 1.3 or were not deregulated in AAV-shA4-treated SCA7 mice anymore (Figure S7C,D; Table S3). Consequently, the numbers of DEGs accounting for the major GO terms associated with disease progression in AAV-shSCR-treated SCA7 mice were reduced by 16% to 47% in AAV-shA4-treated SCA7 mice (Figure 6B), suggesting that AAV-shA4 treatment attenuates the disease burden by preventing the deregulation of these genes. Moreover, among the 140 restored genes, we assigned cell-type-specificity to 82 genes, including 24 PC-type-specific genes (Figure 6C), which were enriched in GO terms related to synapse, dendrite, and ion channel activity and are therefore associated with disease progression (Figure S7E). Furthermore, seven PC-specific genes (Abr, Calb1, Htr1b, Itpr1, Kcnip1, Kcnma1, and Mtss1) were part of the disease signature of downregulated genes involved in the PC pathology shared with SCA1 and SCA2 mice [23]. Finally, another restored PC-type-specific gene, Scn4b, played a role in regulating the repetitive firing properties of mature PCs [27]. Together, these results suggest that the cerebellar pathology, particularly affecting PCs, is mitigated by AAV-shA4 treatment. We noted that 83 genes were deregulated only in AAV-shA4-treated SCA7 mice. However, the analysis of this group of DEGs showed no GO term enrichment (Figure S7F).

## 3. Discussion

To date, there are no effective therapies that significantly modify the course of dominantly inherited CAG/polyQ SCAs. The toxic polyQ SCA proteins trigger a complex cascade of cellular dysfunctions leading to neuronal death. Therefore, preventing this toxic influence by lowering protein expression is currently considered a promising therapeutic approach. However, therapeutic strategies that simultaneously decrease the levels of polyQ-expanded and WT proteins represent a risk for the long-term treatment of patients since the WT SCA proteins have important cellular functions. For instance, ATXN7 is involved in neurodevelopment [28,29,30], and in humans, it can influence the cerebellar volume in healthy adults [31]. To address this limitation, we developed a strategy that targets the disease-causing CAG repeat expansions and that may benefit patients affected by different CAG/polyQ SCAs. To evaluate the therapeutic strategy, we used the SCA7^140Q/5Q^ knock-in mouse model that develops the cerebellar ataxia phenotype in a time frame suitable for preclinical testing. As the mode of delivery, we used the AAV PHP.eB vector that efficiently transduces the cerebellum via non-invasive blood stream injection. Using such a setup, we demonstrated that certain shRNAs are able to target CAG expansions in the cerebellum and to reduce the level of cerebellar mATXN7, however, with a variable degree of allele selectivity and safety profile. We showed that the CAG-targeting shA4 is the most active and allele-selective shRNA and led to the reduction of the mATXN7 cerebellar level by 50%, which had no significant effect on nATXN7. In addition, we showed that the AAV-shA4 treatment of SCA7 mice at an asymptomatic stage resulted in improvement of motor and behavioral phenotypes in the open-field test and was otherwise well tolerated up to 23 weeks post-injection. Finally, AAV-shA4 treatment led to the significant correction of transcriptional alterations in the cerebellum of SCA7 mice.

### 3.1. Variability of Different shRNAs to Safely and Effectively Target CAG Expansion In Vivo

Previous development of the CAG repeat targeting strategy focused primarily on the design of diverse synthetic reagents to optimize allele selectivity and lowering efficiency features. Despite their potency, these synthetic reagents, such as AON, act transiently and would require repeated administration in the clinic to maintain low levels of the polyQ-expanded protein. In contrast, the delivery of shRNA in a single administration of the viral vector provides a valuable strategy for a long-term lowering effect. Given that cellular shRNA processing can give rise to an array of siRNAs of different lengths [14], the allele selectivity of shRNAs designed on the basis of previously active siRNA sequences is difficult to predict and needs to be assessed again in vivo. Indeed, we tested three shRNAs designed from the most allele-selective sd-siRNA sequences (A2R, A4, and A15) reported previously [8], and only shA4 efficiently reduced mATXN7 levels by 50% in the cerebellum of SCA7 mice and had no effect on nATXN7 levels. shA15 had no lowering activity, while shA2R treatment led to a decrease in both mATXN7 and nATXN7 levels (by 38% and 29%, respectively). Interestingly, Kotowska-Zimmer et al. [17] previously reported that A2R shRNA placed in the pri-miR-136 scaffold and delivered by the intra-striatal injection of the AAV5 vector reduced mutant HTT levels by an average of 50% in HD transgenic mice. It is interesting to note that Kotowska-Zimmer’s and our results demonstrate that a single shRNA (shA2R) targeting the CAG expansion can reduce the level of different polyQ-expanded proteins in different disease mouse models following diverse modes of administration. Moreover, both studies reported a certain degree of toxicity following shA2R treatment.

As shA4, shAG4 also selectively lowered mATXN7 levels by 32% in the SCA7 mouse cerebellum. A4 and AG4 antisense sequences exhibited two mismatches with the targeted CAG repeat located at the same nucleotide positions (9 and 15) but involving different bases at position 15 (adenine in A4 versus guanine in AG4). Interestingly, despite a single base difference, shAG4 but not shA4 caused morbidity in mice. shA4(P10,11), as shA2R, also induced morbidity, while shA15 and shSCR did not in our study. Therefore, the morbidity that we observed was likely shRNA sequence-dependent and independent of ATXN7 lowering and may result from off-targeting by shRNA. In particular, we found that shA2R lowered the level of its off-target TH by 55% in the cerebellum of SCA7 mice. The reduction of TH activity or level was previously shown to cause tremor in mice [25,26]. Therefore, further investigation would be necessary to determine whether the reduced TH level is indeed responsible for the tremors in shA2R-treated SCA7 mice. However, it is important to point out that off-targets may differ between mice and humans, as is the case for shA2R not targeting TH in human, prompting caution in interpreting results obtained in non-human models. As for shAG4, given its ability to selectively lower mATXN7 in mice, it would be interesting to further test the AG4 sequence in the pri-miRNA backbone to see whether the toxicity can be avoided while retaining the selectivity of its lowering activity. Therefore, our experimental setup provides a valuable assessment of the safety and ability of these different shRNAs to achieve the targeting of CAG expansion in the mouse cerebellum, and our study constitutes the groundwork to advance toward active reagents for clinical trials.

### 3.2. Attenuation of Ataxia and Cerebellar Phenotypes by CAG-Targeting Strategy

A previous study showed that weekly intra-cerebrovascular injection of chemically modified pure (CUG)7 RNA into SCA3-MJD84.2 and SCA1154Q/2Q mice models resulted in reduced expression of mutant ATXN3 and ATXN1, respectively [16]. However, the effect of this treatment on the ataxia phenotype could not be assessed, possibly due to the very mild or late phenotype of these models. In contrast, the SCA7^140Q/5Q^ knock-in mice readily showed impairment in various motor activity paradigms, including open field tests, which provided a robust readout to test pathology attenuation following treatment [23]. Therefore, we could show that shA4 treatment effectively improved the molecular as well as the behavioral and motor phenotypes of SCA7 mice. Compared to the treatment with the AAV-shSCR control reagent, SCA7 mice treated with AAV-shA4 performed significantly better for several parameters of open field tests, overall demonstrating improved sensori-motor performances and reduced thigmotaxis. AAV-shA4 treatment was also well tolerated up to 23 weeks post-injection and had a very modest effect, per se, on cerebellar transcriptome.

Moreover, shA4 treatment reduced the number of deregulated genes in the SCA7 cerebellum. Restored expression was observed for genes involved in ion channel transport, dendritic formation, and synaptic signaling, which are significant biological pathways previously linked to disease progression in SCA7 mouse cerebellum [23]. In particular, shA4 treatment appeared to attenuate the disease burden of PCs by preventing the downregulation of 24 PC-type-specific genes associated with the synaptic signaling process. It was documented that PCs in SCA7 mice have altered synaptic contact with climbing fibers of inferior olivary neurons and defects in spontaneous firing activity [23] and that many PC identity genes related to synaptic functions are repressed when analyzed in bulk or single-nuclei RNA-seq transcriptomics [23,32]. Interestingly, among the 24 PC genes with restored expression, seven genes (*Abr*, *Calb1*, *Htr1b*, *Itpr1*, *Kcnip1*, *Kcnma1*, and *Mtss1*) were part of a group of 80 PC-type-specific downregulated genes common to the SCA1, SCA2, and SCA7 mouse models and composed an ataxia molecular signature [23]. This signature was notably enriched in genes involved in critical PC functions, such as spontaneous firing, synaptic signaling, and long-term depression [23]. It is noteworthy that *Itpr1*, *Calb1*, *Mtss1*, and *Kcnma1* are known to cause ataxia when mutated in human or mouse models. Mutations in *Itpr1*, which encodes an inositol 1,4,5-trisphosphate receptor involved in calcium release from the endoplasmic reticulum, cause SCA15. Null mutations in *Calb1* (calcium-binding protein) lead to deficits in motor coordination and dendritic calcium signaling in mice [33]. *Kcnma1* encodes the alpha subunit of the large conductance calcium-activated potassium BK channel in PCs, and loss-of-function mutations cause ataxia in mice [34]. *Mtss1*, which encodes an I-BAR containing membrane curving protein involved in the formation of the dendritic arborization of PCs, induces ataxia and elevated levels of activated SFK when mutated in mice [35]. Interestingly, compounds that improve BK channel activity or inhibit SFK activation restore PC firing activity and motor functions in SCA1 mice [36,37]. The genes *Kcnip1* (voltage-gated potassium (Kv) channel-interacting protein) and *Htr1b* (serotonine receptor) play a role in the GABAergic transmission of PCs [38,39], and *Abr* (containing a GTPase-activating protein domain) is involved in cerebellar development [40]. Finally, our transcriptome analysis also identified another PC-type-specific gene, *Scn4b* (sodium channel, type IV, beta), for which the expression level was reduced in the control AAV-shSCR-treated mice and significantly restored in AAV-shA4. Targeted disruption of *Scn4b* in mice results in impaired motor performance and disruption of the high-frequency firing of adult PCs [27]. Therefore, restored expression of this set of genes may contribute to improving the PC activity and motor coordination in SCA7 mice. Taking together all observed benefits of shA4 treatment, our study provides proof of the concept that a CAG repeat-targeting strategy can improve behavioral, motor, as well as molecular phenotypes in a preclinical ataxia mouse model.

### 3.3. Efficacy and Limitation of AAV PHP.eB Serotype for Preclinical Trial in SCA7 Mouse

In a previous study [41], it was shown that different AAV serotypes delivered locally in the cerebellum or systemically via the vasculature can be used to target specific cerebellar cell populations. In the present study, we confirmed that the intravascular systemic injection of the AAV PHP.eB efficiently transduced the PC population based on eGFP fluorescence, while granule neurons, which compose almost 99% of cerebellar neurons, were rarely transduced. Other cells in the molecular layer and deep cerebellar nuclei were also transduced by AAV PHP.eB. In an earlier study of the SCA7 cerebellum, PCs showed the most intense mATXN7 immunolabeling and were morphologically and functionally affected [23]. Since the retro-orbital delivery of AAV PHP.eB shA4 treatment led to a 50% reduction in mATXN7 in the cerebellum of SCA7 mice, we assumed that mATXN7 levels decreased primarily in PCs, which is consistent with restored expression of key PC-specific genes. Further evidence of the efficient transduction of PC population by AAV PHP.eB was provided by the fact that shA2 treatment led to a reduced level of the TH off-target, which is highly expressed only by the PCs in the cerebellum.

Although the cerebellum is a major target for therapeutic development in ataxias, other brain structures are often affected and must also be considered. Recent magnetic resonance imaging of SCA7 mice revealed progressive atrophy of several forebrain regions, including the striatum and hippocampus [42]. We found that systemic injection of AAV PHP.eB efficiently transduced the striatum and hippocampus and led to a reduction of mATXN7 level (by 56% and 39%, respectively) by shA4 treatment. It was previously estimated that around 15% of cells are transduced in these regions by intravascular injection of AAV PHP.eB at an equivalent dose [24]. Therefore, further studies will be needed to determine whether mATXN7 reduction rescues neuronal pathology in these regions and contributes to phenotype improvement.

Photoreceptor degeneration is also a major pathological feature of SCA7 patients and SCA7 knock-in mice [23,43]. As previously reported [44], we also found that AAV PHP.eB was ineffective in transducing retinal photoreceptors in SCA7^140Q/5Q^ mice, and consequently, we could not assess the efficacy of shA4 treatment on visual function. The use of other AAV vector isotypes and/or different delivery routes would be necessary to specifically target retinal photoreceptors in the SCA7^140Q/5Q^ model.

### 3.4. Towards Further Advancement of CAG-Targeting Strategies

In this present study, we showed that systemic administration of shA4 treatment with AAV PHP.eB at the asymptomatic stage in SCA7 mice resulted in a 39–56% reduction of mATXN7 in defined brain regions, including the cerebellum, improved behavioral and motor phenotypes, and restored PC type-specific gene expression. Although the allele discrimination we observed by western blot analysis is promising, given the technical limitations associated with analyses of protein levels in brain tissue, future studies of the extent and persistence of the allele selectivity over time will be needed to establish the basis for progress towards clinical work. In any case, the current results suggest that targeting of the CAG expansion in SCA7 patients, even with a modest decrease in the mutant protein, may provide beneficial improvement in neurological symptoms.

Our results indicate that selective targeting of CAG expansion can be achieved in the context of SCA7^140Q/5Q^ mice, which express a large expansion on the mutated allele and a very short CAG tract on WT. When considering a treatment for SCA7 patients, it should be noted that ATXN7 normal alleles contain relatively short CAG tracts, usually 10 CAGs [20,21]. As the average size of pathogenic alleles is 51 ±13 CAGs, targeting expansion of this range in vivo needs to be demonstrated. On the other hand, it is well known that CAG expansion in the ATXN7 locus is extremely unstable and can increase to >100 CAGs in germline and somatic cells [45]. Therefore, somatic expansion of CAG repeats could be a critical factor in favoring the selectivity of mutant alleles targeting with reagents such as shA4 and shAG4. Further analysis of these reagents in other CAG/polyQ disease models is warranted, in particular because somatic expansions have also been reported.

In the past years, three AONs have been evaluated in clinical trials for HD and have been discontinued. A non-selective AON-lowering total HTT in a Phase III trial (NCT03761849) was halted due to worsening disease status, and two SNP-mediated allele-selective AONs in Phase I/IIa trials (NCT03225833 and NCT03225846) did not lead to a significant reduction of the mutant HTT. More recently, allele-selective AON reagents targeting CAG repeats have progressed to a clinical-stage program for HD, SCA1, and SCA3 (NCT05822908). In this context, our positive results using the AAV-PHP.eB delivery of CAG-targeting shRNA reagents provide a different but promising therapeutic approach for CAG/polyQ SCAs.

## 4. Materials and Methods

### 4.1. Mouse Information

The SCA7^140Q/5Q^ knock-in mice were kept on C57Bl/6J background and bred in the Mouse Clinical Institute (Illkirch, France) with a 12 h light/dark cycle and free access to food and water. Genotyping was performed by PCR as described previously [23]. WT and SCA7 males and females were analyzed (unless indicated), without mixing the genders within the same treatment group. All animal procedures were carried out in strict accordance with the French national laws for laboratory animal welfare and the guidelines of the Federation of European Laboratory Animal Science Associations, based on European Union Legislation (Directive 2010/63/EU). 

### 4.2. Adeno-Associated Virus Production

Recombinant AAV serotype PHP.eB was generated by a triple transfection of the HEK293T/17 cell line using polyethylenimine (PEI) transfection reagent and the 3 following plasmids: pAAV-CMV-eGFP-H1-shRNA (expressing shRNAs targeting CAG repeats or scrambled, sequences given in Figure 1B and Figure 4A), pUCmini-iCAP-PHP.eB [18] (encoding the AAV serotype PHP.eB capsid), and pHelper (Agilent, Santa Clara, CA, USA) (encoding the adenovirus helper functions). After 48 h of transfection, rAAV vectors were harvested from the cell lysate and treated with Benzonase (Merck Millipore 101697, Molsheim, France) at 100 U/mL. They were further purified by gradient ultra-centrifugation with Iodixanol (OptiprepTM density gradient medium, Sigma-Aldrich, Saint-Quentin-Fallavier, France)) followed by dialysis and concentration against Dulbecco’s Phosphate Buffered Saline (DPBS) (Sigma-Aldrich) using centrifugal filters (Amicon Ultra-15 Centrifugal Filter Devices 100K, Merck Millipore). Viral titers were quantified by real-time PCR using the LightCycler480 SYBR Green I Master (Roche Diagnostics, Meylan, France) and primers targeting the eGFP sequence. Titers were expressed as viral genome copies per milliliter (vgc/mL).

### 4.3. Primary Cell Culture and Transfection/Transduction

Mouse fibroblasts were obtained from E13.5 EMbryos of SCA7 heterozygous mice. They were grown for 3 days in DMEM supplemented with 10% FCS and 40 µg/mL of gentamycin. Subsequently, mouse fibroblasts were transduced with AAV-shA2R and AAV-shSCR at two concentrations (MOI 10^5^ or 10^6^) and lysed 6 days post-transduction for a subsequent western blot analysis. Mouse cortical glial cultures were obtained from the post-natal 2-day-old pups of SCA7 heterozygous and homozygous mice and WT littermates. Cultures were grown for 13 days on poly-DL-ornithine-coated plates in DMEM supplemented with 10% FCS, 6 g/L glucose, and 1× Pen/Strep. Oligonucleotides (ONs) were synthesized by Future Synthesis (Poznan, Poland. The sequence of ONs used in this study is as follows: A2/A2M: 5′GCUGCUGCAGCUGCUGCUGCU (A2 is RNA oligonucleotide, whereas A2M contains 5′ Phosphorylation as well as 2′Fluoro-, 2′-O-Methylo- and Phosphorothioate modifications) [8,12]. Glial cell transfections were performed using Lipofectamine 2000 (Invitrogen, Waltham, MA, USA), according to the manufacturer’s instructions. Transfection efficiency was monitored using a control BlockIT fluorescent siRNA (Invitrogen). Cells were lysed 3 days post-transfection for a subsequent WB analysis.

### 4.4. Retro-Orbital Viral Injections in Mice

Before retro-orbital intravenous injections of AAVs, 4.5–5.5 week-old mice were anesthetized by intraperitoneal injection with a mixture of ketamine and xylazine (120/20 mg/kg). They were injected into the right retro-orbital vein sinus with a volume of AAV sample adjusted for the appropriate tested dose and based on mice weight. Untreated SCA7 and WT mice were injected with equivalent volumes of saline solution. The mice from the same experimental group were injected during the same procedure.

### 4.5. Vector Genome Copy Number

Cerebellum, striatum, hippocampus, and liver were lysed in lysis buffer (50 mM Tris pH 8.0, 0.2 M NaCl, 5 mM EDTA, 1% SDS, 0.6 mg/mL proteinase K), and DNA was extracted with phenol-chloroform. Vector genome copy number was measured by quantitative real-time PCR on 5 ng of genomic DNA using the Light Cycler 480 SYBR Green I Master (Roche Diagnostics). The results (vector genome copy number per cell) were expressed as *n*-fold differences in the transgene sequence copy number relative to the copy of the housekeeping mouse TATA-binding protein gene as the internal standard (number of viral genome copies for 2N genome).

### 4.6. eGFP Fluorescence Acquisition

Cryosections: Mice were anesthetized by intraperitoneal injection with a mixture of ketamine and xylazine (100/10 mg/kg) and transcardially perfused with PBS 1x. Brains were immediately dissected, incubated in 4% PFA/PBS 1x overnight, washed 3 times in PBS 1x, cryopreserved with 30% sucrose, mounted in Shandon Cryomatrix embedding resin (Thermo Fisher Scientific, Illkirch-Graffenstaden, France), and immediately frozen. The 14–20 μm brain sagittal cryosections (Leica CM 3050S, Leica, Nanterre, France) were collected and stored at −80 °C. Vibratome sections: Brains were fixed and washed as described above and mounted in 2% agarose/PBS 1x. Vibratome sagittal sections (Leica VT 1000S, Leica) of 50 μm were collected, washed in PBS 1x and then in 20% glycerol/PBS 1x, and stored in 60% glycerol/PBS 1x at −20 °C. The 60% glycerol/PBS 1x was washed out using 20% glycerol/PBS 1x and subsequently PBS 1x before mounting for acquisition. Sections were permeabilized in PBST 1x (PBS 1x/0.1% Triton X-100). Nuclei were counterstained with 1 μg/mL of DAPI (4′,6-diamidino-2-phenylindole dihydrochloride). Slides were mounted with Aqua Poly Mount (Polysciences, Warrington, PA, USA), dried, and stored at +4 °C protected from light. Brain images were acquired either with the Hamamatsu NanoZoomer 2.0 slide scanner (Hamamatsu Photonics, Massy, France) or with a Spinning disc with Leica DMI 8 inverted microscope (Leica) equipped with Hamamatsu Orca flash 4.0 Camera 5 (Gataca Sytem, Massy, France) connected with Metamorph software v7.6 (Molecular Devices, San Jose, CA, USA). For the acquisitions, diode lasers (405, 488 nm) were used. Image brightness and contrast were equivalently adjusted in Fiji software v2.1.0 for display purposes when necessary.

### 4.7. Western Blot Analysis

Brain structures from PBS-perfused animals were dissected and snap frozen in liquid nitrogen. Their whole cell extracts were obtained by the lysis and sonication of the tissue in 150 to 500 µL of the buffer containing 60 mM Tris-Cl (pH 7.5), 2% SDS, 10% sucrose, Protease Inhibitor Cocktail (Roche Diagnostics). Protein concentrations were measured using a BCA assay. A total of 35–60 µg (brain tissue) and 50 µg (cell culture) of total proteins were used for electrophoresis. Electrophoresis was run in NuPAGE™ 3–8% Tris-Acetate Protein Gels in NuPAGE™ Tris-Acetate SDS Running Buffer (Thermo Fisher Scientific). Following electrophoresis, proteins were transferred on nitrocellulose membrane for 2 h at 200 mA. Antibodies were diluted in PBSTM 1x (PBS 1x with 0.1% Tween, and 5% non-fat milk). The following primary antibodies were applied overnight at 4° C: anti-ATXN7, 1:1000 (Thermo Fisher ScientificPA1-749); anti-Vinculin, 1:1000 (Cell Signaling Technology 4650, Danvers, MA, USA); anti-IRF7, 1:5000 (Ozyme 24129S, Saint-Cyr-L’Ecole, France); anti-IFNB, 1: 5000 (Sigma-Aldrich AB2215); anti-TH 1: 1000 (SySy 213102, Synaptic System, Gottingen, Germany); anti-GFAP, 1:1000 (Merck Millipore, MAB3402); anti-ACT1, 1:5000 (IGBMC 2D7, Illkirch, France); anti-GAPDH, 1:10,000 (Merck Millipore MAB374); anti-TUB, 1:2000 (IGBMC 2A2). Secondary antibodies (Peroxidase AffiniPure F(ab’)_2_ Fragment Goat Anti-Rabbit IgG (H + L) (GARPO; 1:10,000, Jackson ImmunoResearch Lab., West Groove, PA, USA), Peroxidase AffiniPure Goat Anti-Mouse IgG (H + L) (GAMPO; 1:10,000, Jackson ImmunoResearch Lab.), and Donkey anti-Goat IgG (H + L) HRP (DAGPO, 1:10,000, Thermo Fisher Scientific) were applied in PBSTM 1x for 1 h at room temperature, washed three times in PBST 1x, and proteins were detected using SuperSignal West Pico PLUS Chemiluminescent kit (Thermo Fisher Scientific). A chemiluminescent signal was acquired on Amersham Imager 600 (Thermo Fisher Scientific). Images were analyzed using Fiji software v2.1.0.

### 4.8. Filter Trap Assay

For the detection of SDS insoluble protein aggregates, samples of a concentration of 5 µg/µL were thawed on ice, vortexed, and 15 µg of protein in 2% SDS in DPBS with 100 mM DTT in a total volume of 100 µL was prepared. Amersham Protran 0.45 µm nitrocellulose membrane (10600002, GE Healthcare, Sigma-Aldrich) was preincubated in DPBS (Thermo Fisher Scientific) with 2% SDS for 5 min and placed into the Minifold^®^ II Slot Blot System (Schleicher & Schuell, GmbH, Dassel, Germany). Wells were washed with 0.1% SDS in DPBS, samples were applied, and wells washed again, once with 0.1% SDS in DPBS and once with DPBS, each after all remaining liquid from the previous step had passed through the membrane. After disassembling the slot blot system, membranes were washed 5 min in TBS and blocked in 5% skim milk powder in TBST for 1 h at RT. To detect mATXN7 aggregates, primary antibody PA1-749 (1:1000 in TBST) was incubated overnight at 4 °C. For fluorescence detection, secondary IRDye antibody goat anti-rabbit 800CW (1:10,000 in TBST, LI-COR Biosciences; Cat# 926-32211, Lincoln, NE, USA) was incubated for 1 h, washed trice with TBST for 30 min, and detected with the LI-COR ODYSSEY^®^ FC (LI-COR Biosciences). Quantification of signal was performed with LI-COR Image Studio Lite software, v4.0.21. Signal intensities were plotted against shSCR treatment.

### 4.9. Open Field Test

The open fields were placed in a room homogeneously illuminated at 150 lux. Mice were tested in automated open fields (44.3 × 44.3 × 16.8 cm, Panlab, Barcelona, Spain) and were virtually divided into central and peripheral regions. Each mouse was placed in the periphery of the open field and allowed to explore freely for 30 min, with the operator outside the test room. The distance traveled, the number of rears, average speeds, and resting time were automatically recorded and reflect the general locomotor or motor activity phenotype. The number of entries and the time spent in the central and peripheral regions were used as an index of emotionality/anxiety [46].

### 4.10. RNA-Seq Library Preparation, Sequencing and Data Analysis

Total RNA samples were extracted from 1 cerebellum/sample using TriReagent (Molecular Research Center, Cincinnati, OH, USA) following the manufacturer’s instruction. RNA-Seq libraries were generated from 200 ng of total RNA using Illumina Stranded mRNA Prep, a Ligation kit, and IDT for Illumina RNA UD Indexes Ligation, according to manufacturer’s instructions (Illumina, San Diego, CA, USA). Briefly, Oligo(dT) magnetic beads were used to purify and capture the mRNA molecules containing polyA tails. The purified mRNAs were then fragmented at 94 °C for 8 min and copied into first strand complementary DNA (cDNA) using reverse transcriptase and random primers. Second strand cDNA synthesis further generated blunt-ended double-stranded cDNA and incorporated dTTP in place of dUTP to achieve strand specificity by quenching the second strand during amplification. Following A-tailing of DNA fragments and ligation of pre-index anchors, PCR amplification was used to add indexes and primer sequences and to enrich DNA libraries (30 s at 98 °C [10 s at 98 °C, 30 s at 60 °C, 30 s at 72 °C] × 13 cycles; 5 min at 72 °C). Surplus PCR primers were further removed by purification using SPRIselect beads (Beckman-Coulter, Villepinte, France), and the final libraries were checked for quality and quantified using capillary electrophoresis. Libraries were sequenced on an Illumina Hiseq 4000 sequencer as single-read 50 bases following Illumina’s instructions.

Image analysis and base calling were performed using RTA 2.7.7 and bcl2fastq version 2.20.0.422. According to Illumina Stranded mRNA Prep Ligation—Reference Guide—PN 1000000124518, the first base of each read was trimmed. Reads were preprocessed using cutadapt version 1.10 [47] to remove adapter, polyA, and low-quality sequences (Phred quality score below 20); reads shorter than 40 bases were discarded for further analysis. Reads mapping to rRNA were also discarded (this mapping was performed using bowtie version 2.2.8 [48]). Reads were then mapped onto mm10 mouse genome assembly using STAR version 2.5.3a [49]. Quantification of gene expression was performed using HTSeq v0.6.1p1 [50] and gene annotations from Ensembl release 102. Differential gene expression analysis was performed using R version 3.3.2 and DESeq2 version 1.16.1 Bioconductor library [51]. *p*-values were adjusted for multiple tests using the Benjamini and Hochberg method [52]. Gene expression values (indicated as reads per kilobase (RPK) throughout the Section 2) correspond to read counts normalized across libraries with the method proposed by [53] (size factors computed using DESeq2 v1.16.1 Bioconductor library) and divided by gene length (calculated as the median of the length of all transcripts corresponding to this gene in kb). Genes were considered expressed only when RPK ≥ 1 as a mean of WT samples. Gene functional annotations were performed using ShinyGO v0.80 (http://ge-lab.org/go/, accessed on 19 January 2023) [54].

### 4.11. Experimental Design and Statistical Analysis

Data were analyzed using GraphPad Prism 8 and R. For a comparison of two groups, data were tested for normality using Shapiro’s test. Differences between means were assessed with unpaired Student’s *t* test, one-way ANOVA, or ordinary two-way ANOVA followed by post-hoc Tukey’s test testing of pairwise comparisons among genotypes and treatments. Significance was established at *p* < 0.05. Data are expressed as mean ± SEM unless indicated. Further information is indicated in the figure legends. Datasets used and/or analyzed during the current study are available from the corresponding author upon reasonable request.

## Data Availability

The RNA-seq data were deposited in NCBI Gene Expression Omnibus (http://www.ncbi.nlm.nih.gov/geo/ (accessed on 10 October 2023)) and are accessible through accession number GSE244970. Other data that support the findings of this study are available from the corresponding authors on reasonable request.

## Supplementary Materials

The following supporting information can be downloaded at: https://www.mdpi.com/article/10.3390/ijms25084354/s1.
