# Supplementary material for: AAV-Mediated CAG-Targeting Selectively Reduces Polyglutamine-Expanded Protein and Attenuates Disease Phenotypes in a Spinocerebellar Ataxia Mouse Model
Source: Int J Mol Sci. 2024 Apr 15;25(8):4354. doi: 10.3390/ijms25084354 (PMC11050704; doi:10.3390/ijms25084354)
Supplement: Supplementary file 1 [file ijms-25-04354-s001.zip › Figures S1-S8_Niewiadomska_Cimicka.pdf]

AAV-mediated CAG-targeting selectively reduces polyglutamine-expanded protein and attenuates disease phenotypes in a spinocerebellar ataxia mouse model

Anna Niewiadomska-Cimicka [1*], Lorraine Fievet [1], Magdalena Surdyka [2], Ewelina Jesion [2], Celine Keime [1], Elisabeth Singer [3,4,5], Aurelie Eisenmann [1], Zaneta Kalinowska-Poska [2], Hoa Huu Phuc Nguyen [5], Agnieszka Fiszer [6], Maciej Figiel [2], Yvon Trottier [1*]

1. Institute of Genetics and Molecular and Cellular Biology, INSERM U1258, CNRS UMR7104, University of Strasbourg, Illkirch, France.
2. Department of Molecular Neurobiology, Institute of Bioorganic Chemistry Polish Academy of Sciences, Poznan, Poland.
3. Centre for Rare Diseases (ZSE), University of Tuebingen, D-72076 Tuebingen, Germany.
4. Institute of Medical Genetics and Applied Genomics, University of Tuebingen, D-72076 Tuebingen, Germany.
5. Department of Human Genetics, Medical Faculty, Ruhr University Bochum, Bochum 44801, Germany.
6. Department of Medical Biotechnology, Institute of Bioorganic Chemistry, Polish Academy of Sciences, Noskowskiego 12/14, Poznan, Poland.

SUPPLEMENTARY FIGURES S1-S8

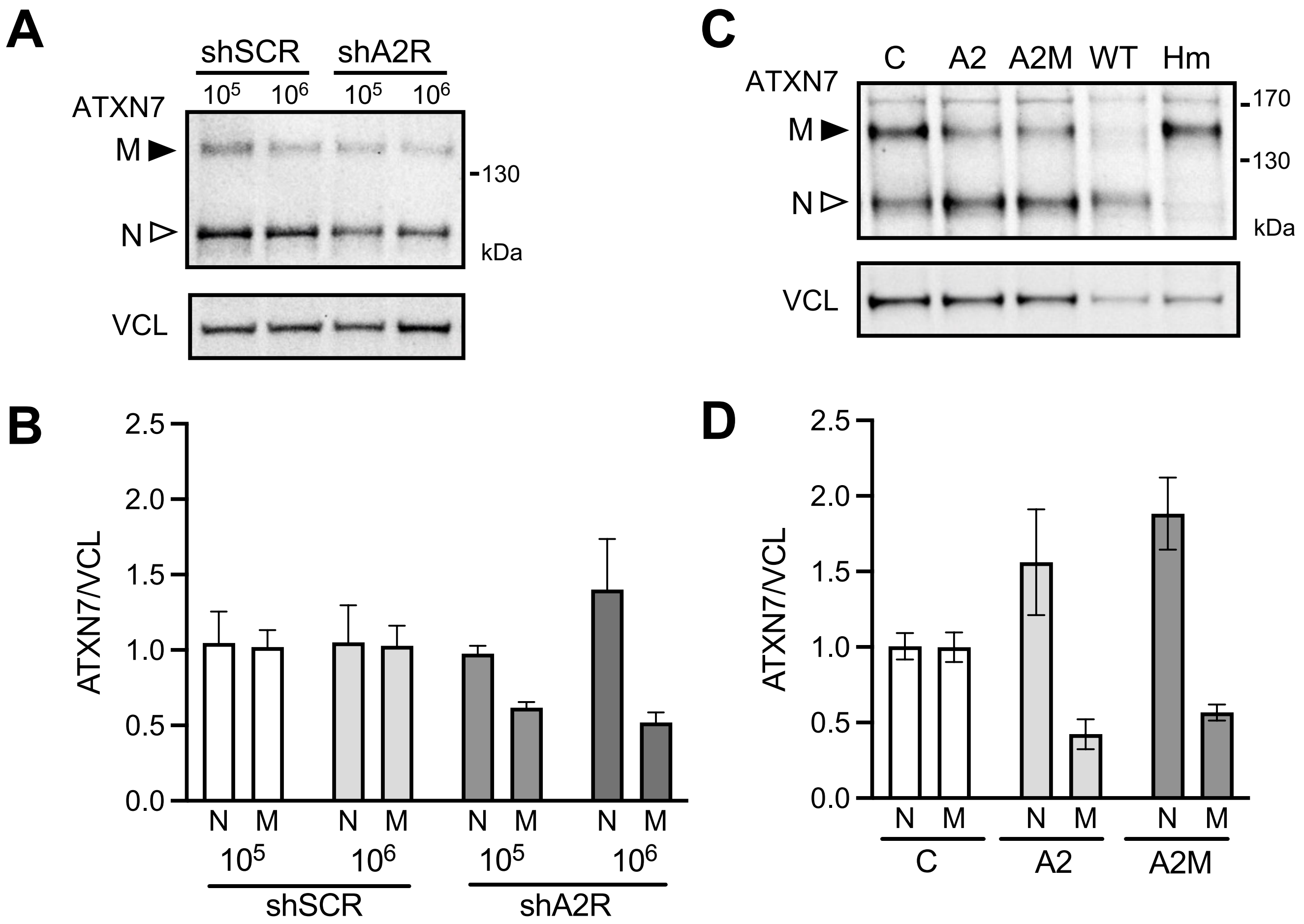

Supplementary Figure S1. Lowering of mATXN7 by A2R shRNA, sd-siRNAs A2 and A2M in cultured primary cells of SCA7 mice.

(A) Western blot analysis of normal (N) and mutant (M) ATXN7, and Vinculin (VCL) in primary mouse embryonic fibroblasts 6 days after transduction with AAV-shA2R and AAV-shSCR at two concentrations (MOI 10^5 or 10^6).

(B) Signal intensities of ATXN7 protein alleles in (A) normalized to VCL levels. Graph is plotted relative to shSCR (at MOI 10^5 or 10^6), with mean set at 1. Bars are mean ± SEM (four technical replicates).

(C) Western blot analysis of ATXN7 and VCL levels in primary glial cells 72 h after transfection of 100 nM A2 and A2M sd-siRNA and control siRNA (C). Cell cultures samples obtained from WT and SCA7140/140Q homozygous (Hm) mice are used as controls, respectively, for nATXN7 and mATXN7 protein migration on blots.

(D) Signal intensities of ATXN7 protein alleles in (C) normalized to VCL levels. Graph is plotted relative to control siRNA, with mean set at 1. Bars are mean ± SEM (four technical replicates). Note that while A2 and A2M led to a decrease of mATXN7, they also caused an increase of nATXN7 level, as previously reported for the normal human ATXN7 protein in SCA7 patient fibroblasts (Fiszer et al., 2016b).

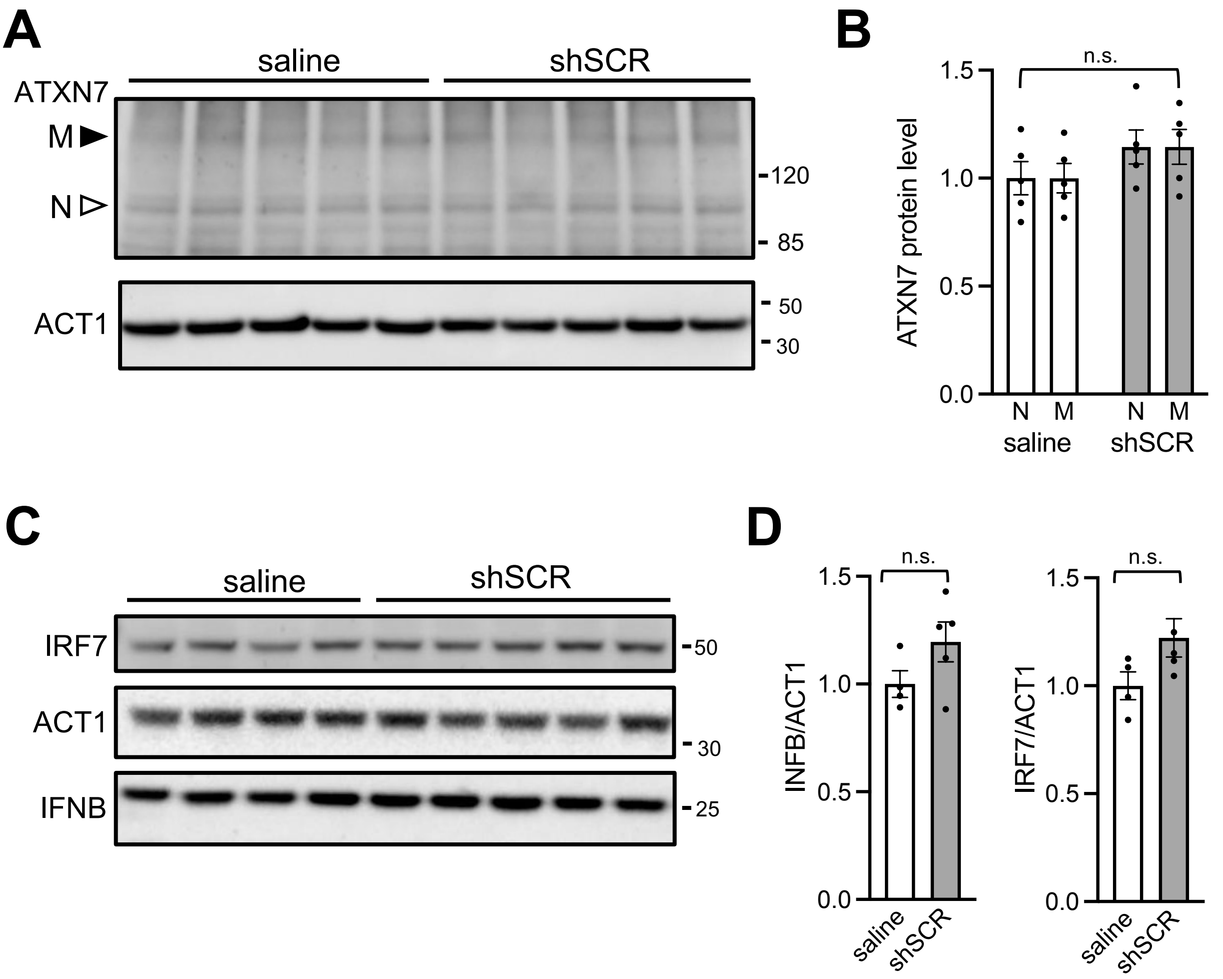

Supplementary Figure S2. Lack of ATXN7 lowering effect and immune system activation after injection of AAV-shSCR in mice.

(A) Western blot analysis of normal (N) and mutant (M) ATXN7, and Actin-1 (ACT1) in cerebellar samples of SCA7 mice injected with saline or with AAV-shRNA (3.0 x 10^{13} vg/kg) at the age of 4.5-5.5 weeks and sacrificed at 9 weeks.

(B) Signal intensities of ATXN7 normalized to ACT1 levels.

(C) Western blot analysis of Interferon Regulatory Factor 7 (IRF7), Interferon Beta (INFB) and ACT1.

(D) Signal intensities of IRF7 and INFB normalized to ACT1 levels.

Graphs are plotted relative to saline controls with mean set at 1. Data are expressed as mean ±SEM and analyzed using two-tailed Student's *t*-test. n.s., not significant ($p > 0.05$).

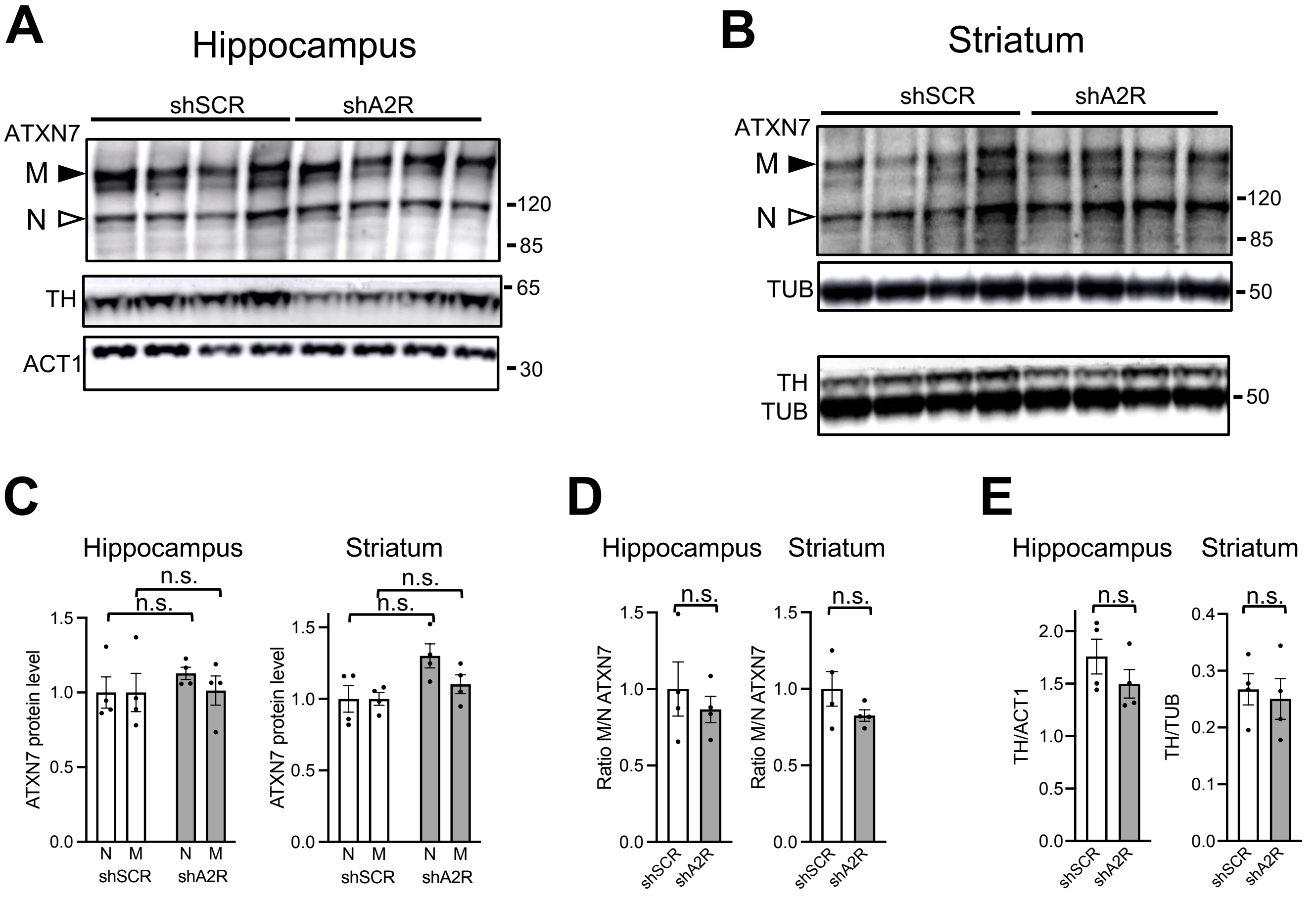

Supplementary Figure S3. No effect on ATXN7 and TH expression in hippocampus and striatum after injection of AAV-shA2R in SCA7 mice.

(A, B) Representative western blot analysis of ATXN7, TH, ACT1 and TUB in the hippocampus and striatum of SCA7 mice injected with at $1.5x10^{13}$ vg/kg with AAV-shA2R or AAV-shSCR. Mice were injected at age of 6 weeks and sacrificed at 10 weeks post-injection for analysis.

(C) Signal intensities of normal (N) and mutant (M) ATXN7 in the hippocampus and striatum, normalized to ACT1 and TUB levels, respectively.

(D) Ratio of signal intensities of normalized mATXN7/nATXN7 levels.

(E) Signal intensities of TH in the hippocampus and striatum, normalized to ACT1 and TUB levels, respectively.

Graphs in (C) and (D) are plotted relative to shSCR conditions with mean set at 1. Data are expressed as mean ±SEM and analyzed using two-tailed Student's *t*-test. n.s. for not significant

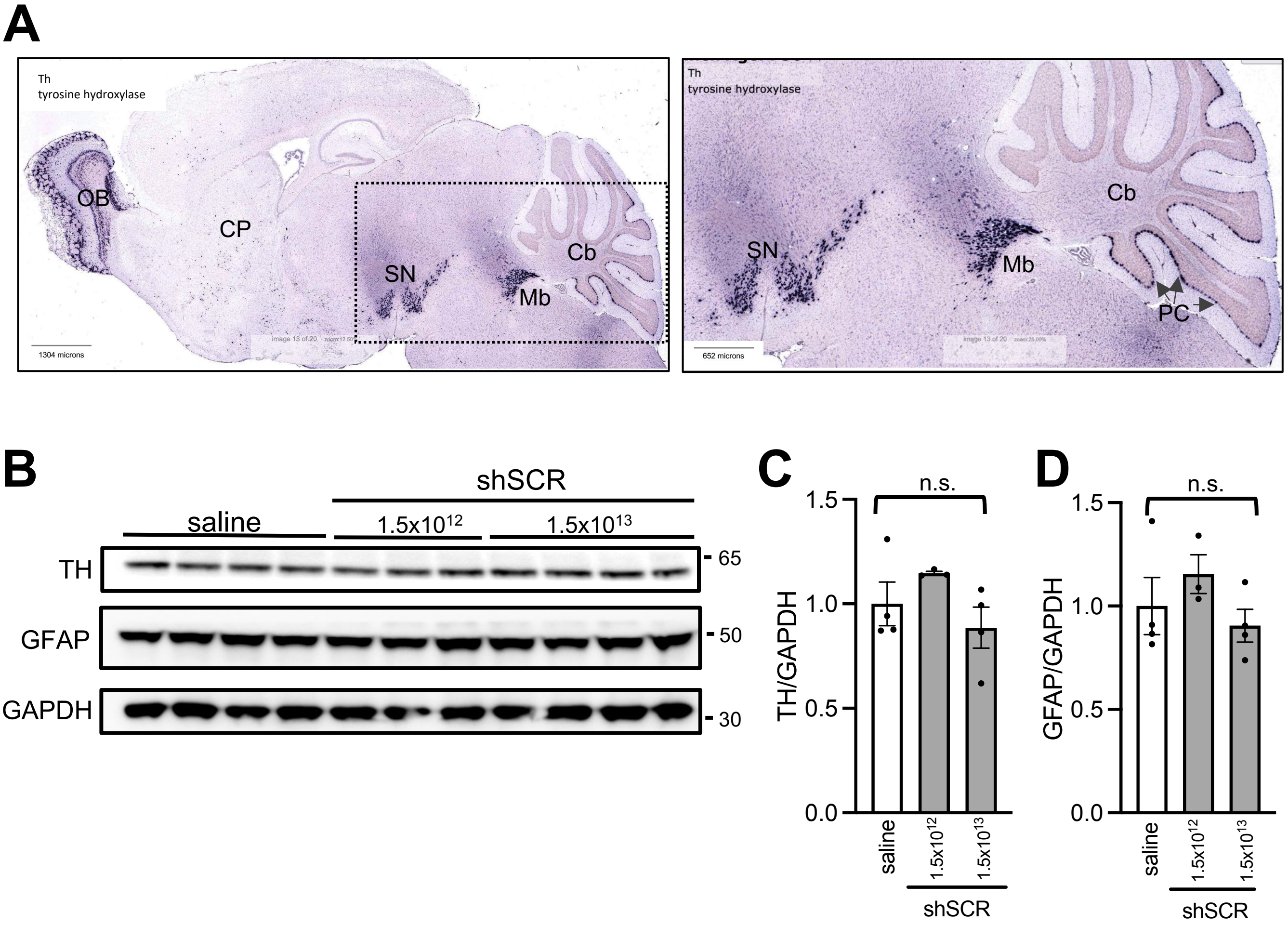

Supplementary Figure S4. Th brain expression and protein analysis of TH and GFAP in AAV-shSCR in mice.

(A) In situ hybridization of Th probe in adult mouse brain (Lein et al., 2007). Allen mouse brain atlas: mouse.brain-map.org/gene/show/21582

(B) Western blot analysis of Glial Fibrillary Acidic Protein (GFAP), Tyrosine Hydroxylase (TH), and GAPDH in cerebellar samples of SCA7 mice injected with saline or with AAV-shSCR at different doses (vg/kg).

(C) Signal intensities of TH normalized to GAPDH levels.

(D) Signal intensities of GFAP normalized to GAPDH levels.

Note that compared to saline, AAV-shSCR induces neither a decrease in TH level, nor an increased level of the gliosis marker GFAP. Graphs are plotted relative to saline conditions with mean set at 1. Data are expressed as mean ± SEM and analyzed using two-tailed Student's t-test. n.s., not significant ($p > 0.05$).

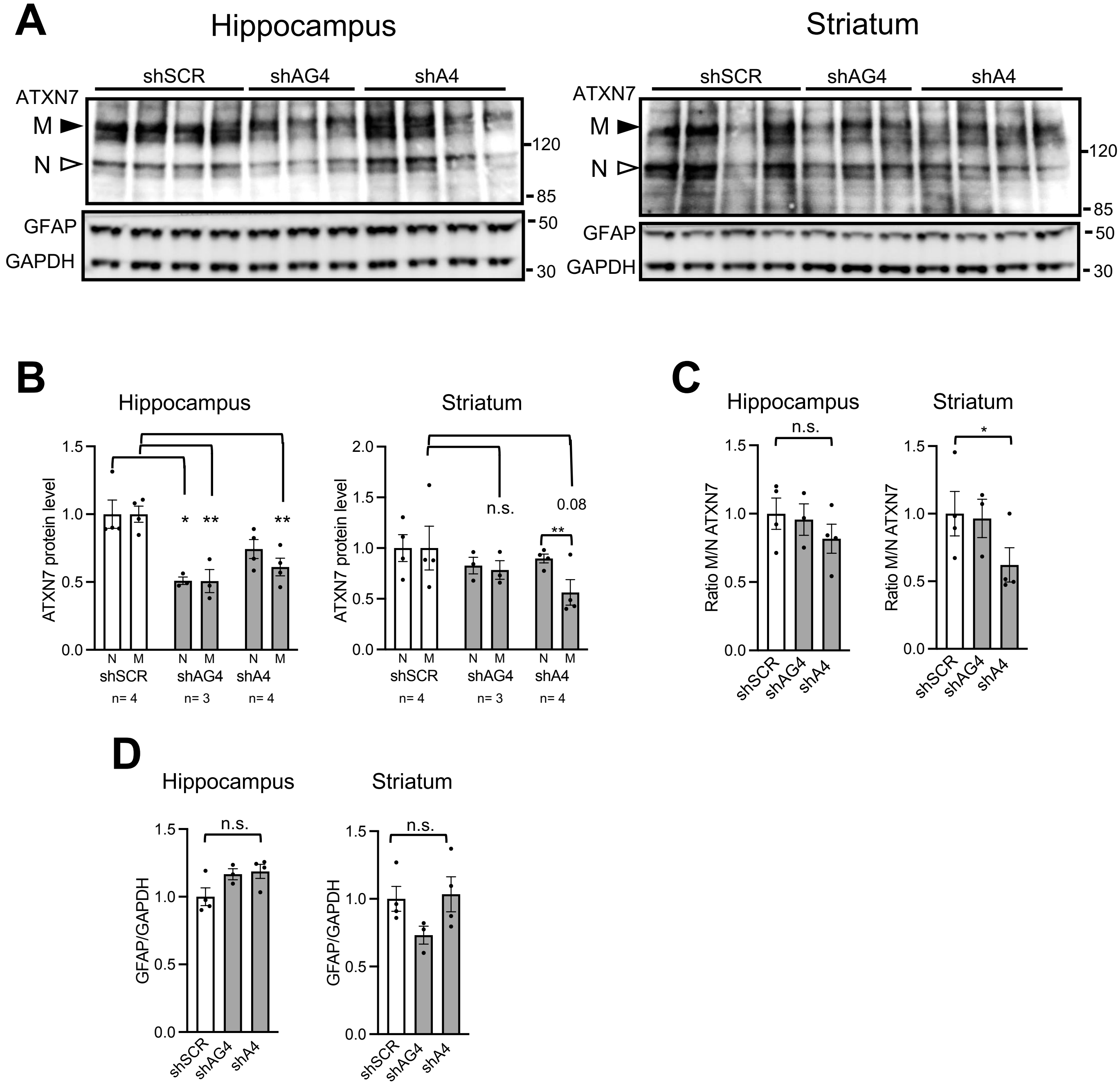

Supplementary Figure S5. Analysis of the efficacy, allele-selectivity and safety of AAV-shA4 and AAV-shAG4 in the hippocampus and striatum of SCA7 mice.

(A) Representative western blot analysis of ATXN7, GFAP and GAPDH in the hippocampus and striatum of SCA7 mice injected with at $1.5x10^{13}$ vg/kg with AAV-shA4, AAV-shAG4 or AAV-shSCR. Mice were injected at age of 5 weeks and sacrificed at 7 weeks post-injection for analysis.

(B) Signal intensities of normal (N) and mutant (M) ATXN7 normalized to GAPDH levels and plotted relative to SCR conditions. One striatal sample (value =0.95) was identified as outlier using Grubb method, and was excluded from statistical analysis.

(C) Ratio of signal intensities of mATXN7/nATXN7 normalized to GAPDH levels.

(D) Signal intensities of GFAP normalized to GAPDH levels and plotted relative to SCR conditions.

Data are expressed as mean ±SEM and analyzed using two-tailed Student's *t*-test. * $p < 0.05$; ** $p < 0.01$. n.s. for not significant

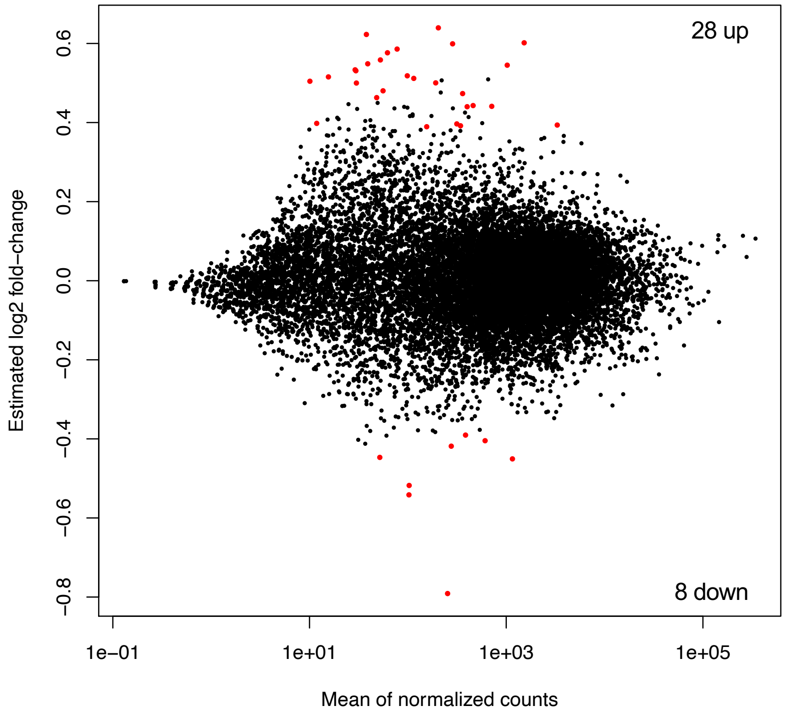

Supplementary Figure S6. Effect of AAV-shA4 treatment on the cerebellar transcriptome in WT mice.

MA plot of transcriptomic dataset between AAV-shA4-treated WT mice (n= 3 males) and untreated WT mice (n= 3 males) at 9 week post injection. This figure represents the estimated log2 fold-change as a function of the mean of normalized read counts, for all protein coding genes with mean of WT RPK > 1. Among those genes, red dots correspond to genes with |log2 fold-change| > 0.385 and adjusted p-value < 0.05.

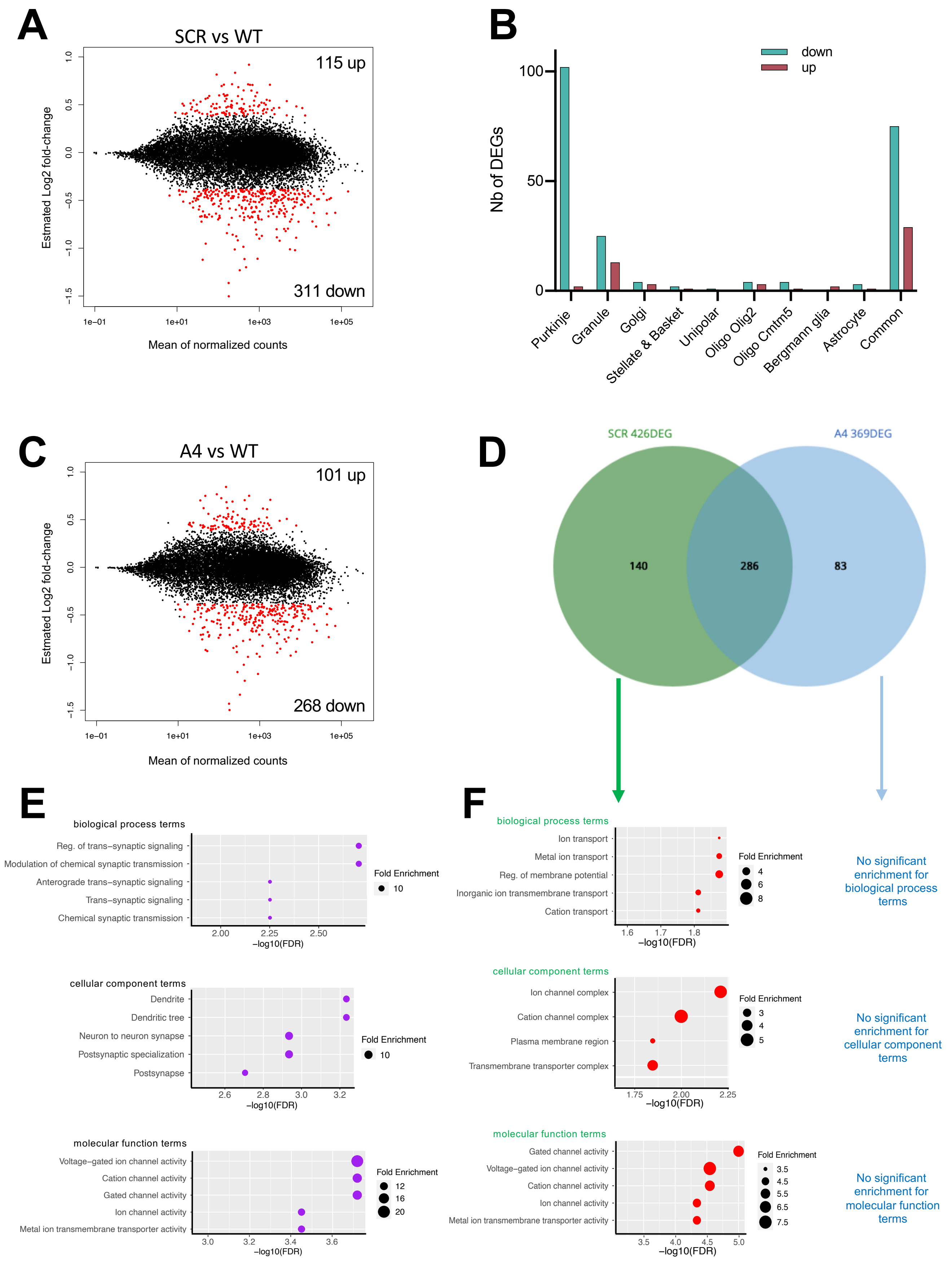
A
SCR vs WT
115 up
311 down
Estmated Log2 fold-change
Mean of normalized counts
B
Nb of DEGs
down
up
Purkinje
Granule
Golgi
Stellate & Basket
Unipolar
Oligo Olig2
Oligo Cmtm5
Bergmann glia
Astrocyte
Common
C
A4 vs WT
101 up
268 down
Estmated Log2 fold-change
Mean of normalized counts
D
SCR 426DEG
A4 369DEG
140
286
83
E
biological process terms
Reg. of trans−synaptic signaling
Modulation of chemical synaptic transmission
Anterograde trans−synaptic signaling
Trans−synaptic signaling
Chemical synaptic transmission
Fold Enrichment
−log10(FDR)
cellular component terms
Dendrite
Dendritic tree
Neuron to neuron synapse
Postsynaptic specialization
Postsynapse
Fold Enrichment
−log10(FDR)
molecular function terms
Voltage−gated ion channel activity
Cation channel activity
Gated channel activity
Ion channel activity
Metal ion transmembrane transporter activity
Fold Enrichment
−log10(FDR)
F
biological process terms
Ion transport
Metal ion transport
Reg. of membrane potential
Inorganic ion transmembrane transport
Cation transport
Fold Enrichment
−log10(FDR)
No significant enrichment for biological process terms
cellular component terms
Ion channel complex
Cation channel complex
Plasma membrane region
Transmembrane transporter complex
Fold Enrichment
−log10(FDR)
No significant enrichment for cellular component terms
molecular function terms
Gated channel activity
Voltage−gated ion channel activity
Cation channel activity
Ion channel activity
Metal ion transmembrane transporter activity
Fold Enrichment
−log10(FDR)
No significant enrichment for molecular function terms

Supplementary Figure S7. Analysis of differentially expressed genes in the cerebellum of AAV-shSCR- and AAV-shA4-treated SCA7 mice compared to WT mice.

(A) MA plot of transcriptomic dataset between AAV-shSCR-treated SCA7 mice (n= 5 males) at 28 weeks of age (23 weeks post-injection) and age-matched WT mice (n= 4 males). This figure represents the estimated log2 fold-change as a function of the mean of normalized read counts, for all protein coding genes with mean of WT RPK > 1. Among those genes, red dots correspond to genes with |log2 fold-change| > 0.385 and adjusted p-value < 0.05.

(B) Cell type-specific distribution of DEGs in AAV-shSCR-treated SCA7 mice as compared to age-matched WT mice. Among the 426 DEGs, 269 genes were assigned to one or more cerebellar cell types.

(C) MA plot of transcriptomic dataset between AAV-shA4-treated SCA7 mice (n= 5 males) at 28 weeks of age (23 weeks post-injection) and age-matched WT mice (n= 4 males). This figure represents the estimated log2 fold-change as a function of the mean of normalized read counts, for all protein coding genes with mean of WT RPK > 1. Among those genes, red dots correspond to genes with |log2 fold-change| > 0.385 and adjusted p-value < 0.05.

(D) Venn diagram showing the overlap of DEGs in the cerebellum of AAV-shSCR- and AAV-shA4-treated SCA7 mice.

(E) Gene ontology (GO) term enrichment, as calculated with ShinyGO 0.77, for the 24 PC type-specific genes that are downregulated in AAV-shSCR, but are restored to a FC < 1.3 or are not deregulated in AAV-shA4-treated SCA7 mice anymore. The 5 most enriched GO terms of each category are sorted by –Log10(FDR).

(F) Gene ontology (GO) term enrichment for the 140 genes only significantly deregulated in AAV-shSCR-treated mice, and 83 genes only deregulated in in AAV-shA4 treated mice, calculated with ShinyGO 0.77. The 5 most enriched GO terms of each category are sorted by –Log10(FDR).

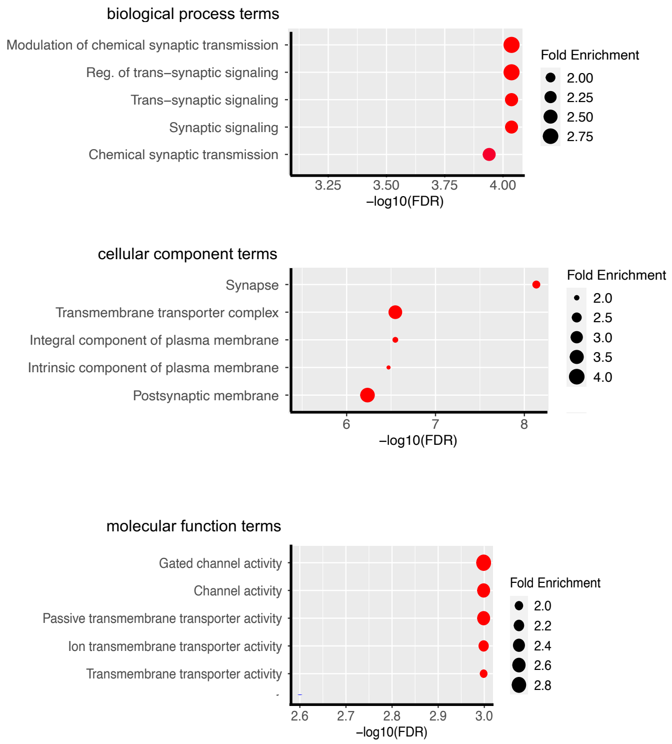

Supplementary Figure S8. Gene deregulation in SCA7 mice at 40 weeks old.
Gene ontology (GO) terms of 466 DEGs (adjusted $p < 0.05$, $|Log2FC| > 0.385$) (Niewiadomska-Cimicka et al., 2021) calculated with ShinyGO 0.8. The 5 most enriched GO terms of each category are sorted by –Log10(FDR).
