# Supplementary material for: AAV-Mediated CAG-Targeting Selectively Reduces Polyglutamine-Expanded Protein and Attenuates Disease Phenotypes in a Spinocerebellar Ataxia Mouse Model
Source: Int J Mol Sci. 2024 Apr 15;25(8):4354. doi: 10.3390/ijms25084354 (PMC11050704; doi:10.3390/ijms25084354)
Supplement: Supplementary file 1 [file ijms-25-04354-s001.zip › Table S1_Niewiadomska_Cimicka.pdf]

Supplementary Table S1: Potential off-targets of CAG targeting shRNAs.

Reagent	Gene name	Description	Length (nt)*	Off-target level**	Brain Expression#	Other tissues#
**A2**	*Ccdc177*	Coiled-coil domain containing 177	21	1	very low	very low
	Maml1	Mastermind like transcriptional coactivator 1	21	1	low	broad & low
	Ppp1r3f	Protein phosphatase 1 regulatory subunit 3F	21	1	medium	very low
	Golga4	Golgin A4	20	1	very low	broad & low
	Soga3	SOGA family member 3	20	1	very low	very low
	Th	Tyrosine hydroxylase	20	1	specific & high	very low
	Mink1	Misshapen like kinase 1	20	2	high	broad & high
**A4**	*Stc1*	Stanniocalcin 1	21	1	very low	specific & medium
	A2230006KO3Rik		20	1		
	Pum1	Pumilio RNA binding family member 1	20	1	medium	broad & medium
	D6Ertd527e		20	2		
	Mad1l1	Mitotic arrest deficient 1 like 1	20	2	very low	broad and low
	Tbl1x	Transducin beta like 1 X-linked	20	2	very low	broad & low
	Zfp853	Zinc Finger Protein 853	20	2	low	broad & low
**A15**	*none*					
**AG4**	*Ccdc177*	Coiled-coil domain containing 177	20	2	very low	very low
	Lrch3	Leucine rich repeats and calponin homology domain containing 3	20	2	low	broad & low
	Maml1	Mastermind like transcriptional coactivator 1	20	2	low	broad & low
	Ppp1r3f	Protein phosphatase 1 regulatory subunit 3F	20	2	medium	very low
	Stc1	Stanniocalcin 1	20	2	very low	specific & medium
**A4$_{(P10,11A)}$**	*Kmt2d*	Lysine methyltransferase 2D	20	2	low	broad & low

* Number of matched nucleotides out of 21, with cut-off 19 and less.
** According to Blast bit-score, with level 1 > 40, and level 2 between 34 to 40.
# Expression source : www.gtexportal.org
