# Supplementary material for: AAV-Mediated CAG-Targeting Selectively Reduces Polyglutamine-Expanded Protein and Attenuates Disease Phenotypes in a Spinocerebellar Ataxia Mouse Model
Source: Int J Mol Sci. 2024 Apr 15;25(8):4354. doi: 10.3390/ijms25084354 (PMC11050704; doi:10.3390/ijms25084354)
Supplement: Supplementary file 1 [file ijms-25-04354-s001.zip › Uncropped Western blots.pdf]

AAV-mediated CAG-targeting selectively reduces polyglutamine-expanded protein and attenuates disease phenotypes in a spinocerebellar ataxia mouse model

Anna Niewiadomska-Cimicka [1*], Lorraine Fievet [1], Magdalena Surdyka [2], Ewelina Jesion [2], Celine Keime [1], Elisabeth Singer [3,4,5], Aurelie Eisenmann [1], Zaneta Kalinowska-Poska [2], Hoa Huu Phuc Nguyen [5], Agnieszka Fiszer [6], Maciej Figiel [2], Yvon Trottier [1*]

1. Institute of Genetics and Molecular and Cellular Biology, INSERM U1258, CNRS UMR7104, University of Strasbourg, Illkirch, France.
2. Department of Molecular Neurobiology, Institute of Bioorganic Chemistry Polish Academy of Sciences, Poznan, Poland.
3. Centre for Rare Diseases (ZSE), University of Tuebingen, D-72076 Tuebingen, Germany.
4. Institute of Medical Genetics and Applied Genomics, University of Tuebingen, D-72076 Tuebingen, Germany.
5. Department of Human Genetics, Medical Faculty, Ruhr University Bochum, Bochum 44801, Germany.
6. Department of Medical Biotechnology, Institute of Bioorganic Chemistry, Polish Academy of Sciences, Noskowskiego 12/14, Poznan, Poland.

SUPPLEMENTARY FIGURES S9-S19
(uncropped western blots)

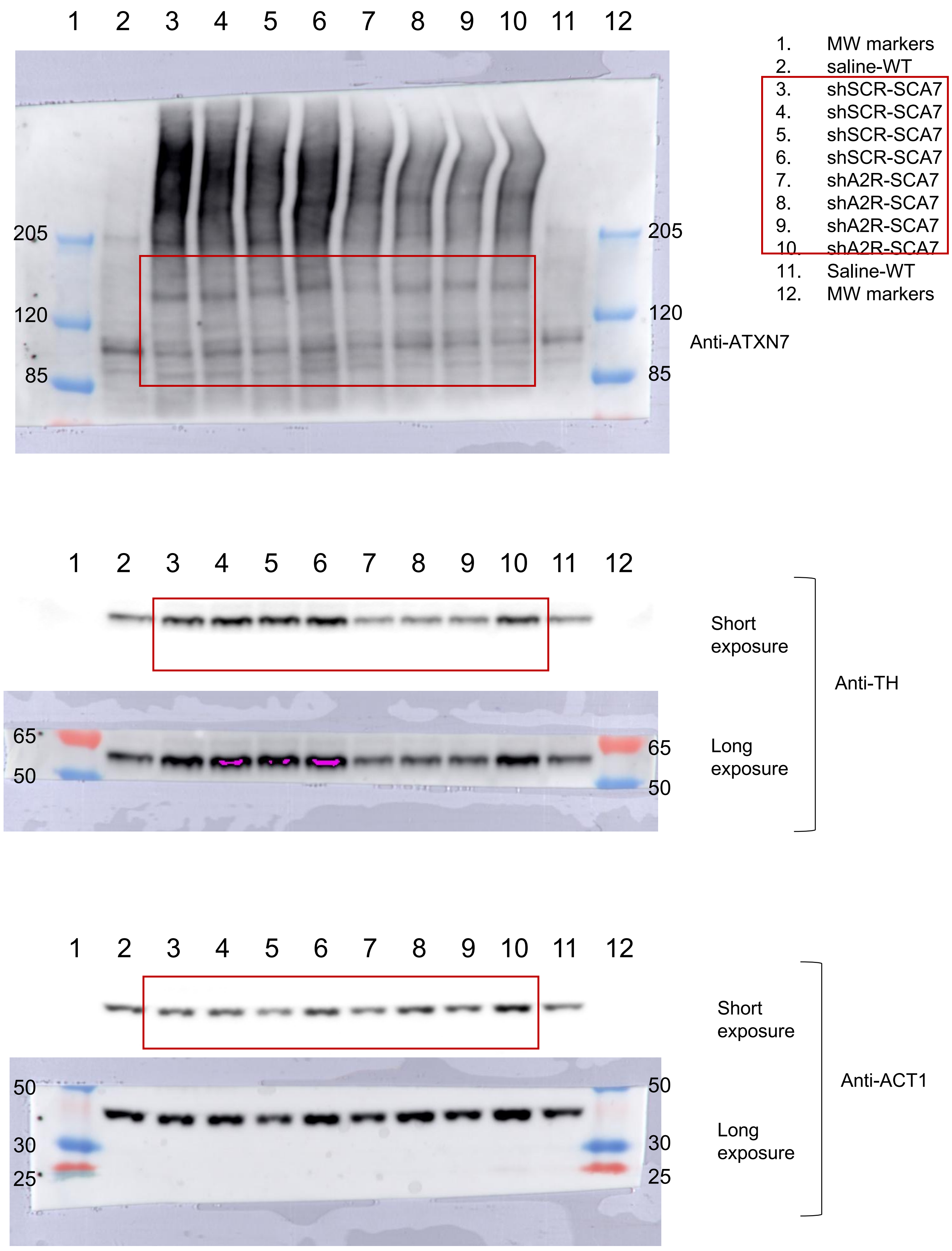

Supplementary Figure S9. Uncropped western blot images of Fig. 3A showing ATXN7, TH and ACT1 expression in the cerebellum.
Note: one western blot membrane was cut in three horizontal pieces for three immunodetections.

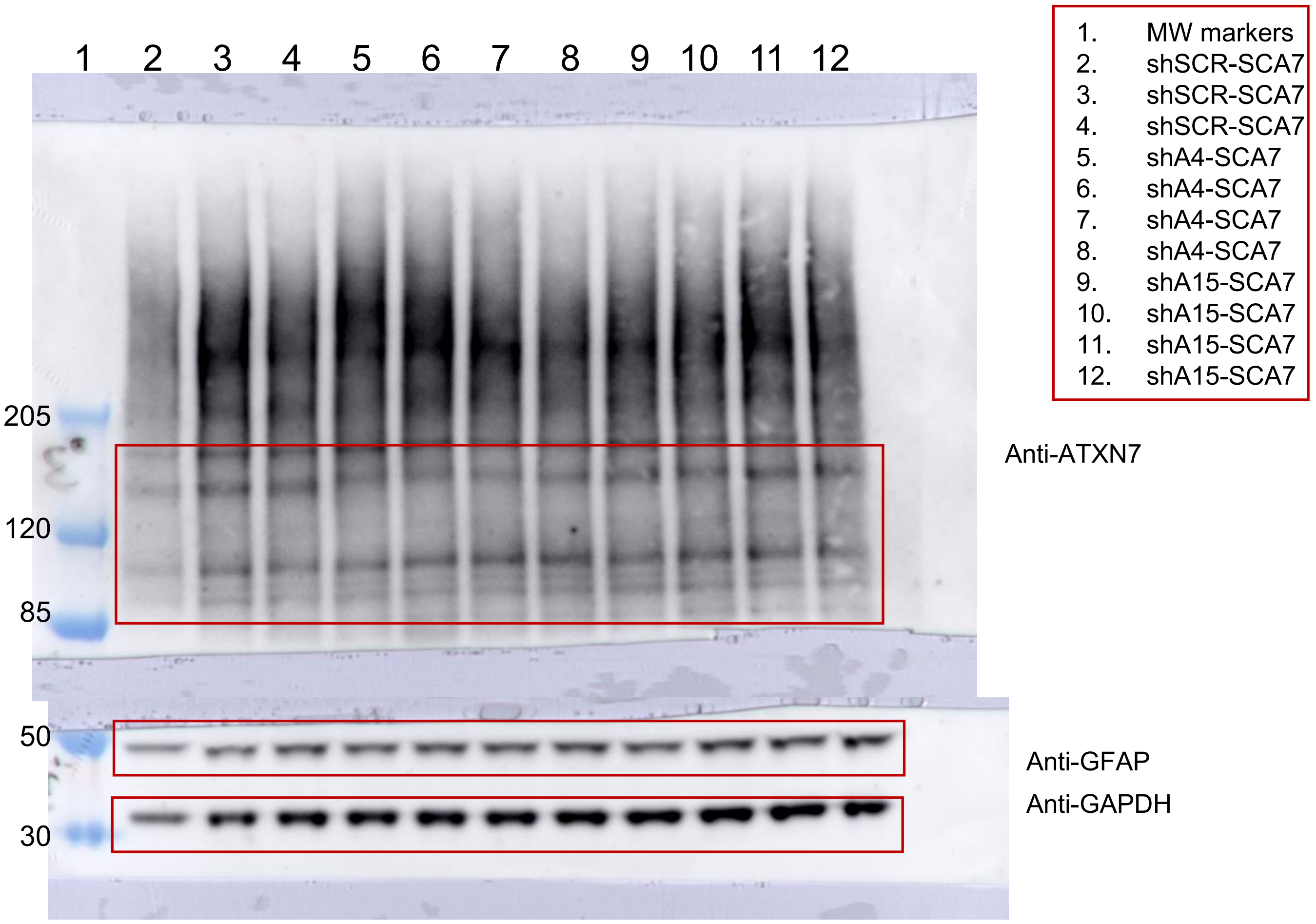

Supplementary Figure S10. Uncropped western blot images of Fig. 4B left panel showing ATXN7, GFAP and GAPDH expression in the cerebellum.

Note: one western blot membrane was cut in two horizontal pieces for three immunodetections.

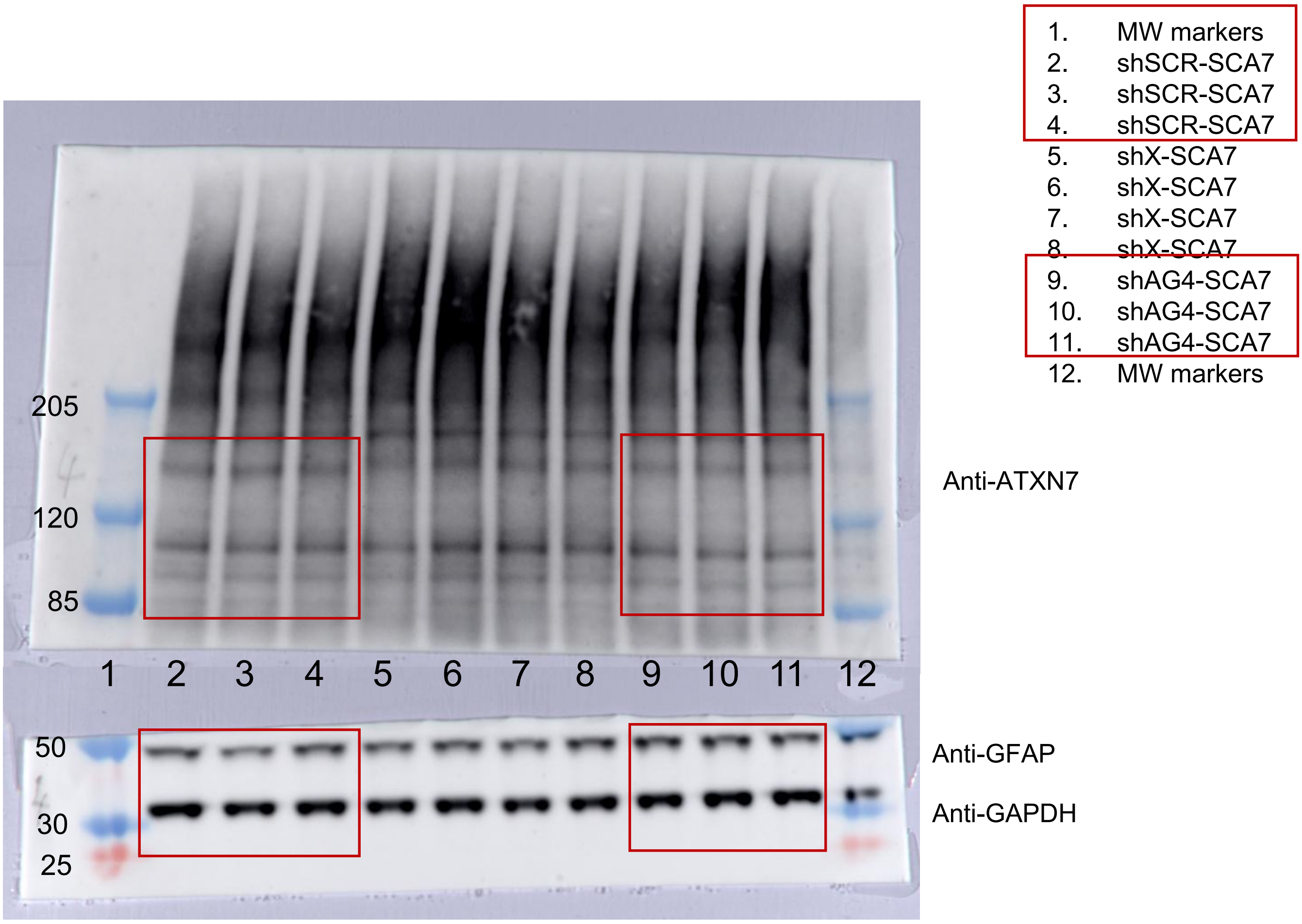

Supplementary Figure S11. Uncropped western blot images of Fig. 4B middle panel showing ATXN7, GFAP and GAPDH expression in the cerebellum.
Note: one western blot membrane was cut in two horizontal pieces for three immunodetections.

1. MW markers saline-SCA7
2. saline-SCA7
3. saline-SCA7
4. saline-SCA7
5. saline-SCA7
6. shSCR-SCA7
7. shSCR-SCA7
8. shSCR-SCA7
9. shSCR-SCA7
10. shSCR-SCA7
11. shA4(P10,11A)-SCA7
12. shA4(P10,11A)-SCA7
13. shA4(P10,11A)-SCA7
14. shA4(P10,11A)-SCA7
15. shA4(P10,11A)-SCA7

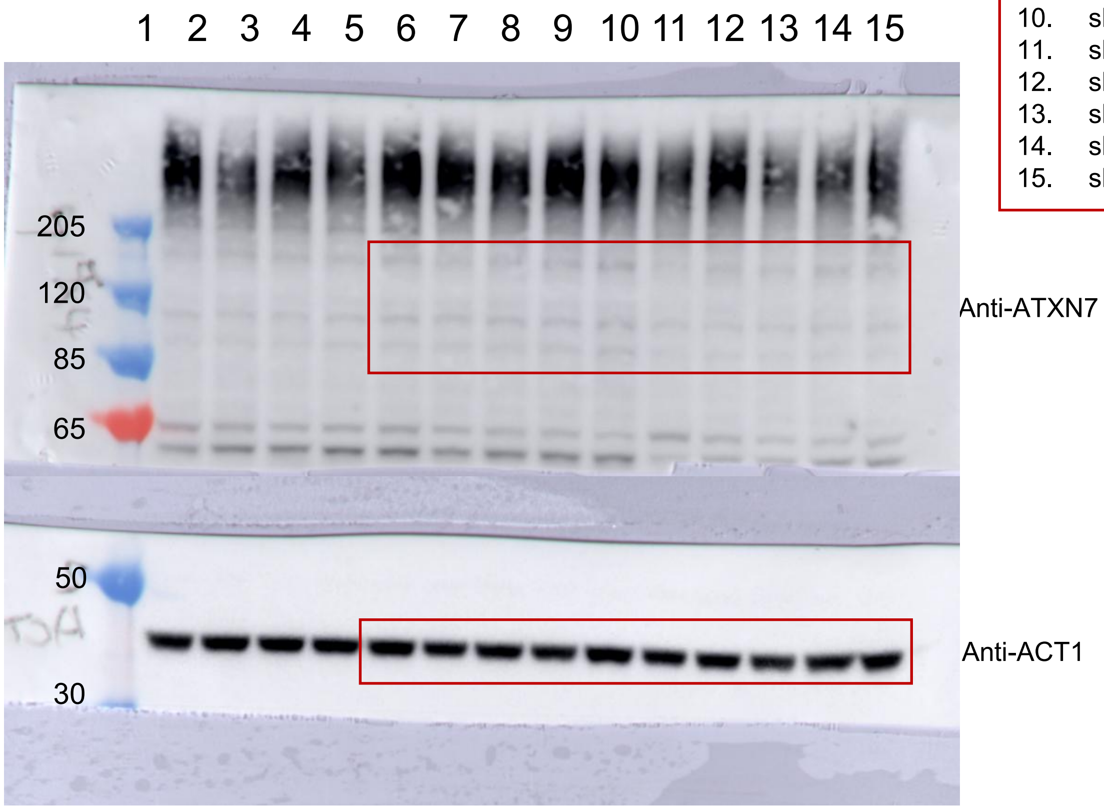

Supplementary Figure S12. Uncropped western blot images of Fig. 4B right panel showing ATXN7 and ACT1 expression in the cerebellum.

Note: one western blot membrane was cut in two horizontal pieces for two immunodetections.

1. MW markers saline-SCA7
2. saline
3. saline
4. saline
5. saline
6. saline
7. shSCR-SCA7
8. shSCR-SCA7
9. shSCR-SCA7
10. shSCR-SCA7
11. shSCR-SCA7
12. saline

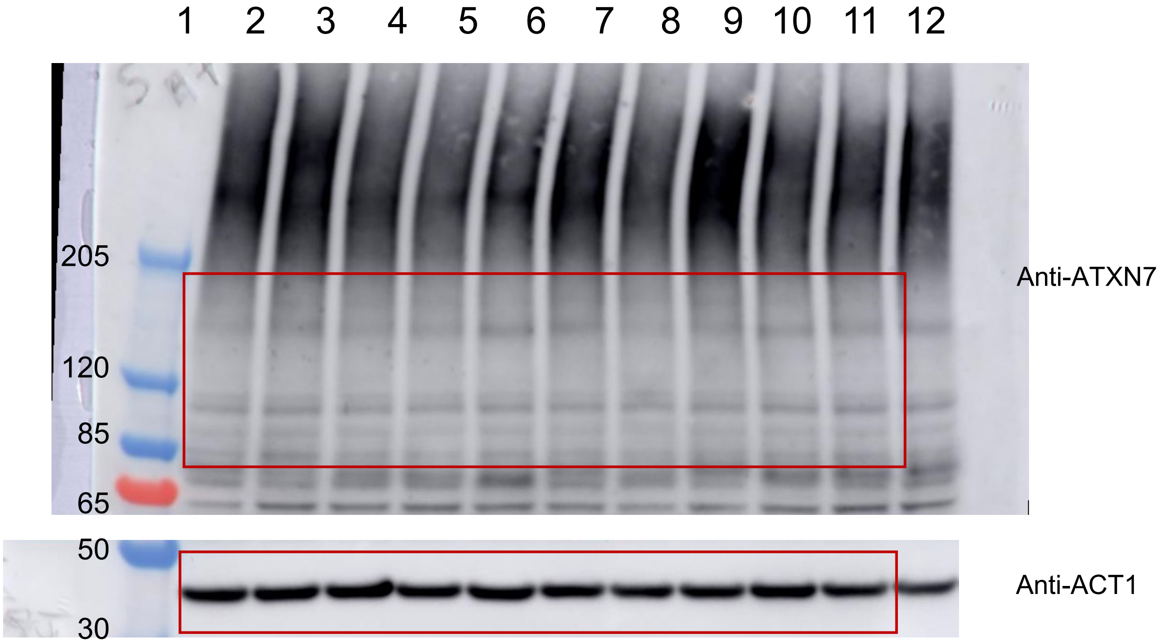

Supplementary Figure S13. Uncropped western blot images of Suppl. Fig. S2A showing ATXN7, and ACT1 expression in the cerebellum.

Note: one western blot membrane was cut in two horizontal pieces for two immunodetections.

1. MW markers saline-SCA7
2. Saline-SCA7
3. Saline-SCA7
4. Saline-SCA7
5. Saline-SCA7
6. shSCR-SCA7
7. shSCR-SCA7
8. shSCR-SCA7
9. shSCR-SCA7
10. shSCR-SCA7
11. shX-SCA7
12. shX-SCA7
13. shX-SCA7
14. shX-SCA7
15. shX-SCA7

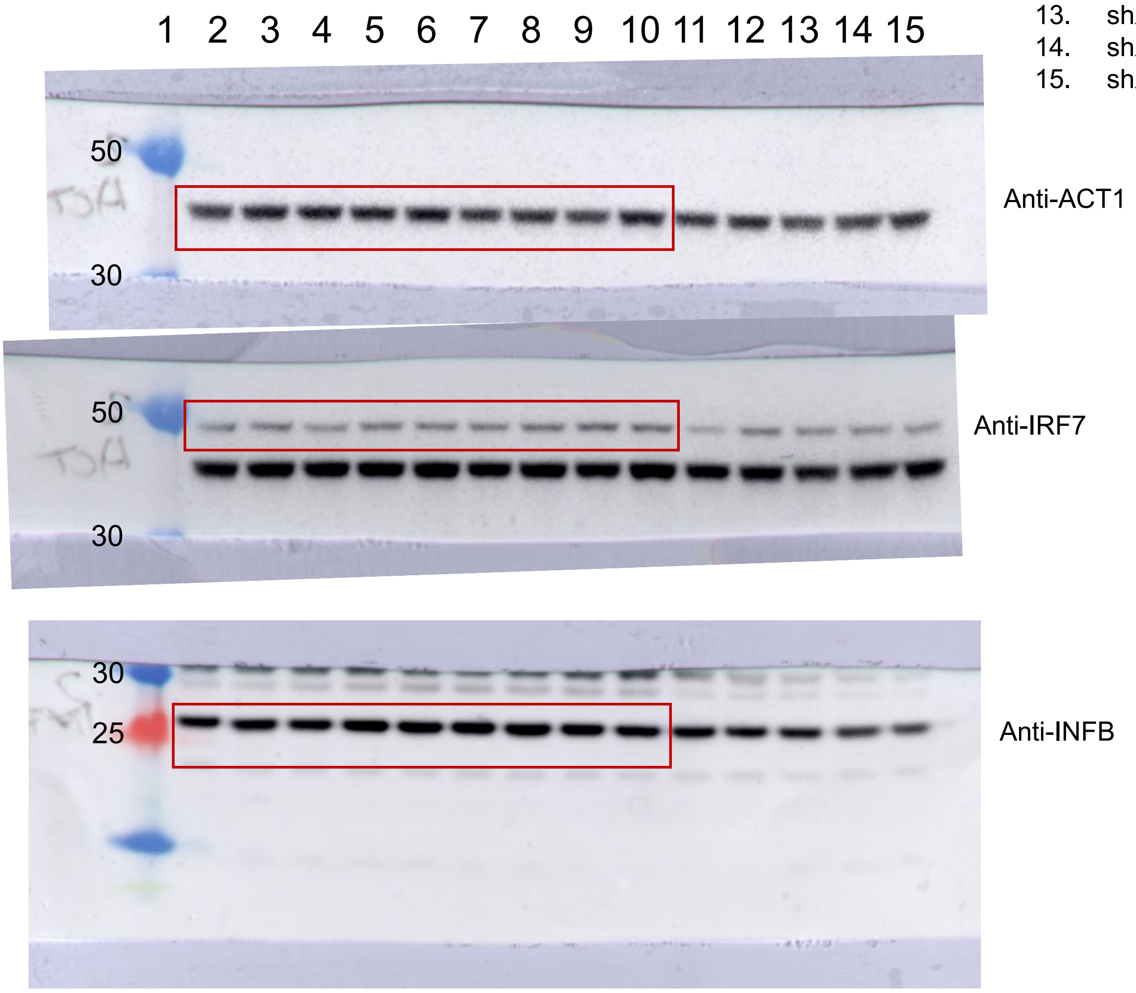

Supplementary Figure S14. Uncropped western blot images of Suppl. Fig. S2C showing ACT1, IRF7 and INFB expression in the cerebellum.
Note: one western blot membrane was cut in two horizontal pieces for three immunodetections.

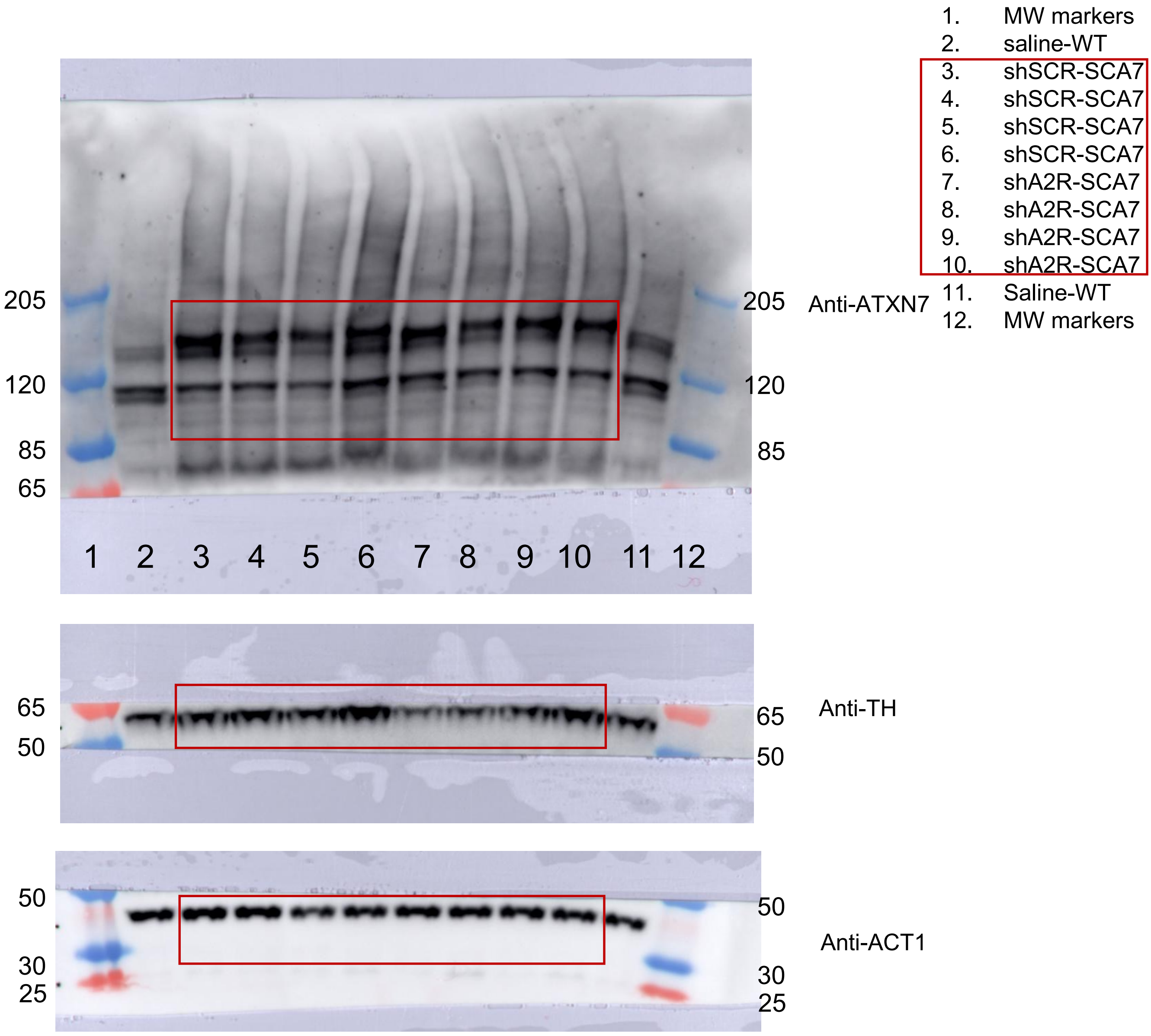

Supplementary Figure S15. Uncropped western blot images of Suppl. Fig. S3A showing ATXN7, TH and ACT1 expression in the hippocampus.

Note: one western blot membrane was cut in three horizontal pieces for three immunodetections.

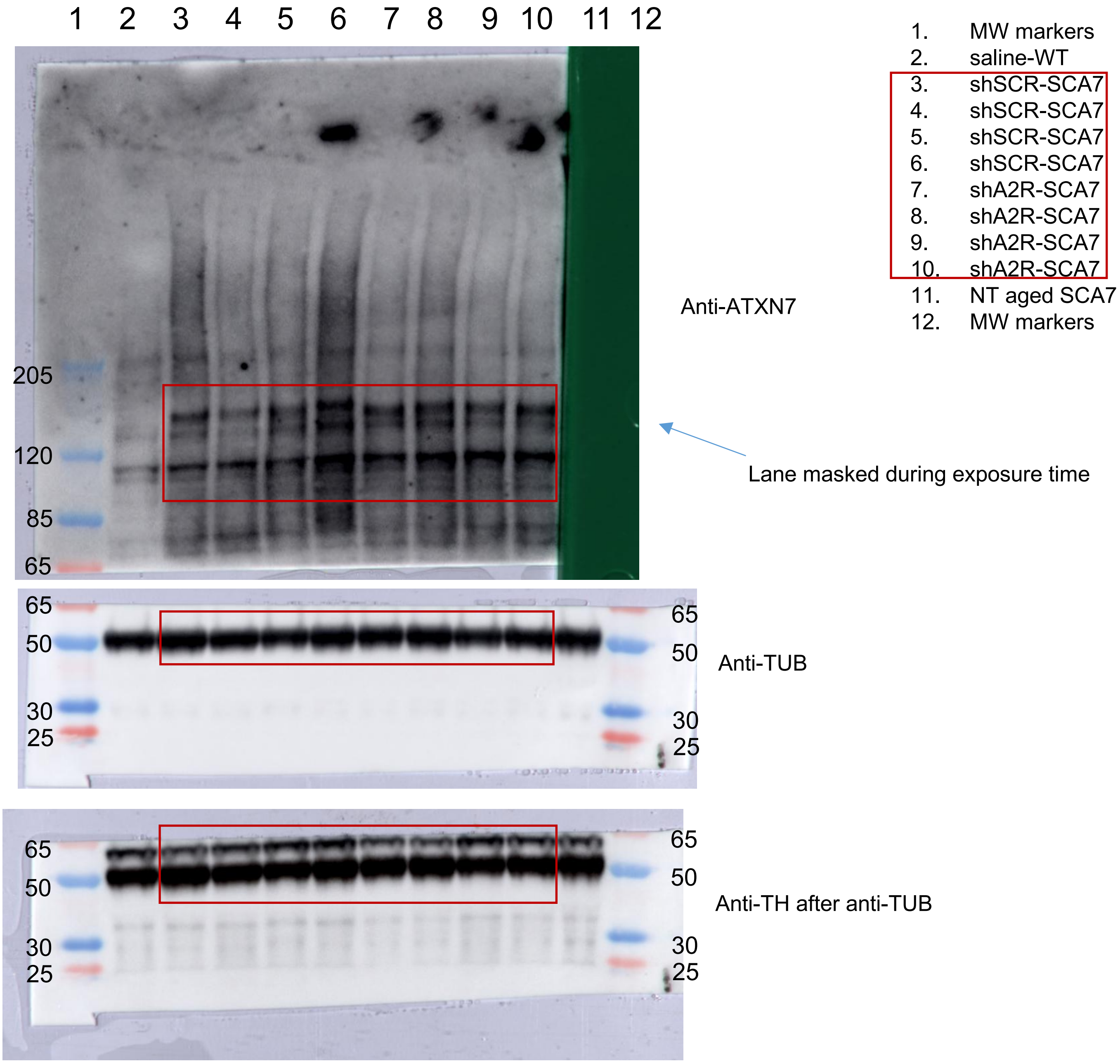

Supplementary Figure S16. Uncropped western blot images of Suppl. Fig. S3B showing ATXN7, TUB and TH expression in the striatum.
Note: one western blot membrane was cut in two horizontal pieces for three immunodetections.

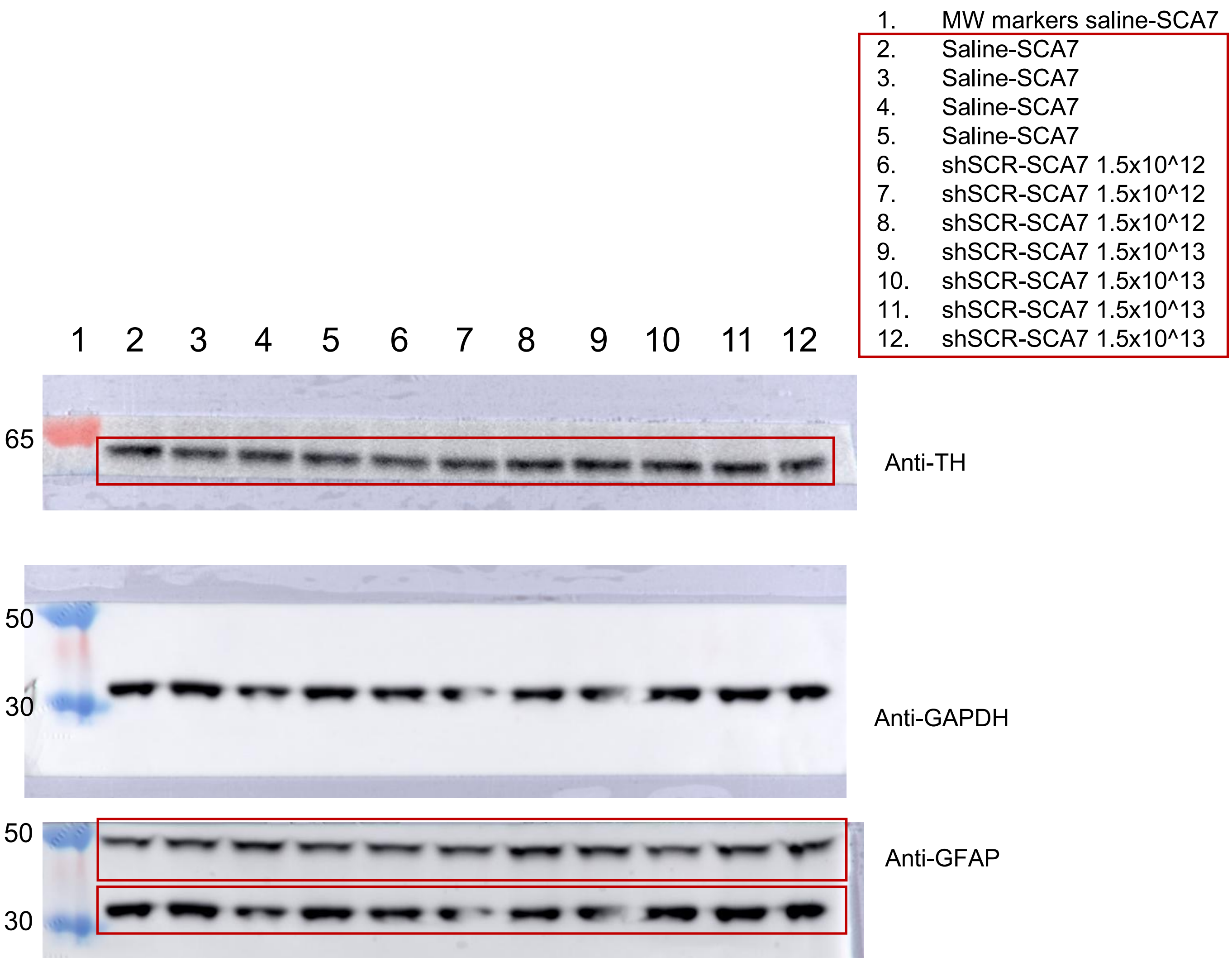

Supplementary Figure S17. Uncropped western blot images of Suppl. Fig. S4B showing TH, GFAP and GAPDH expression in the cerebellum.
Note: one western blot membrane was cut in three horizontal pieces for three immunodetections.

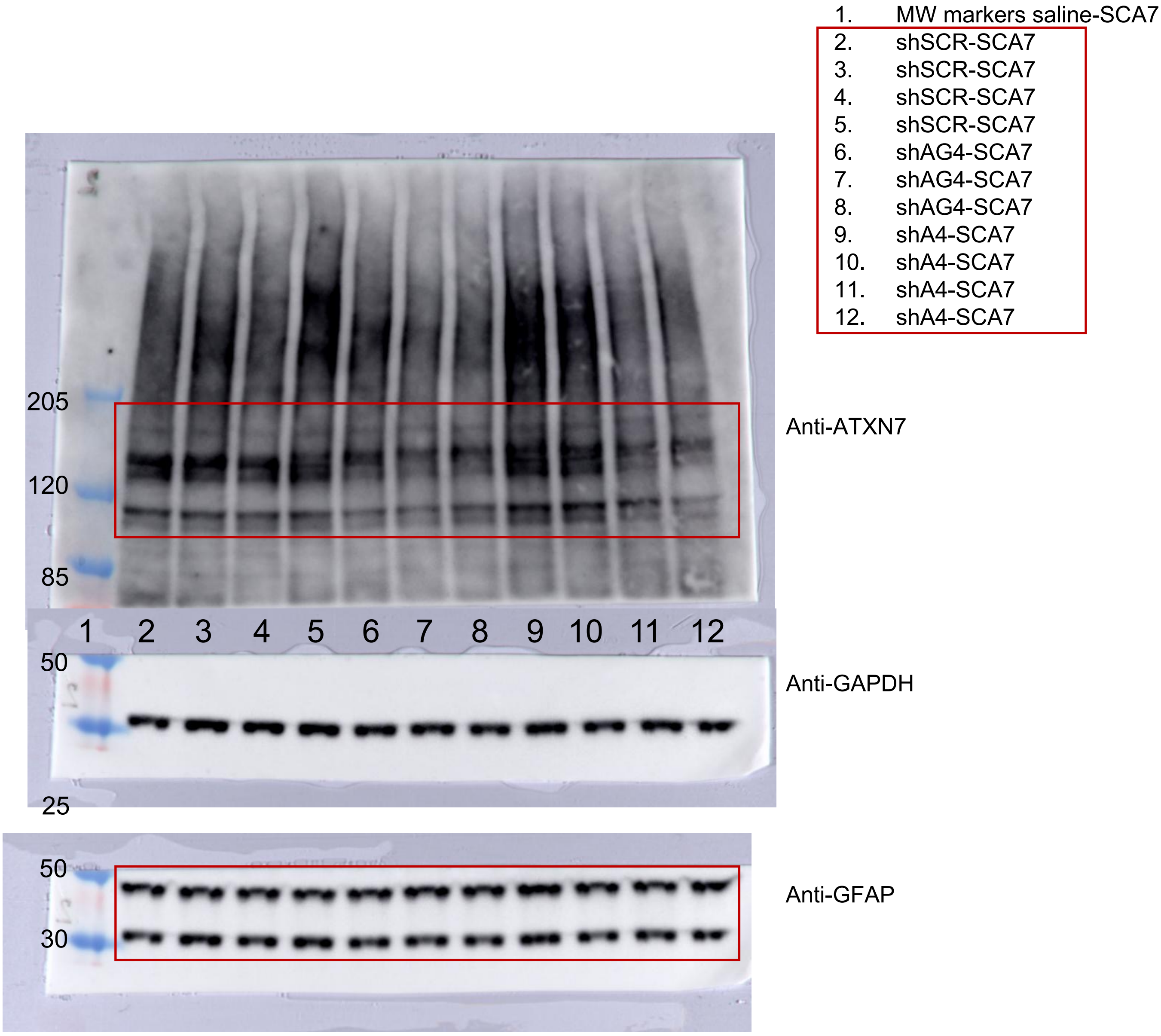

Supplementary Figure S18. Uncropped western blot images of Suppl. Fig. S5 left panel showing ATXN7, GFAP and GAPDH expression in the hippocampus.
Note: one western blot membrane was cut in two horizontal pieces for three immunodetections.

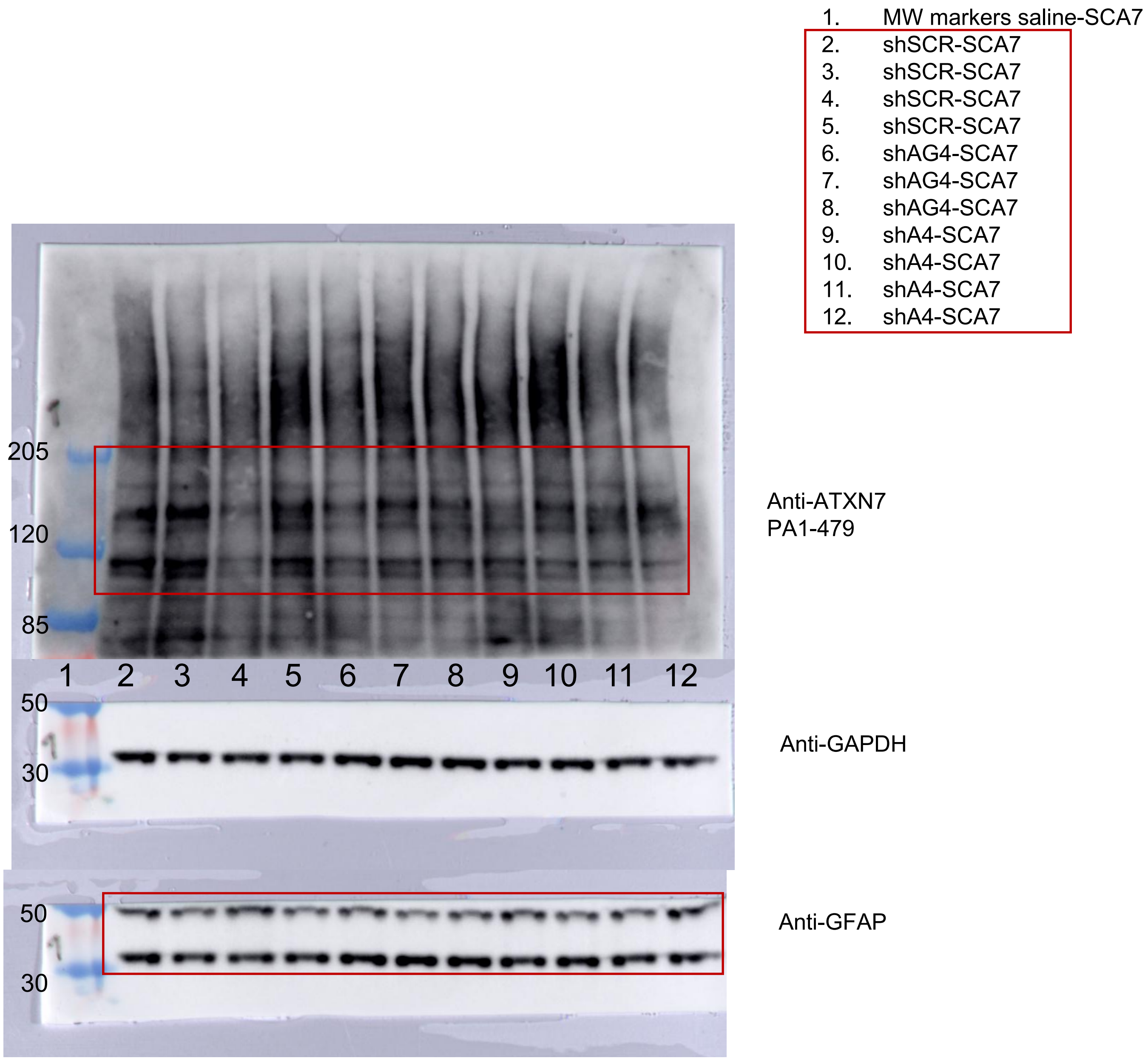

Supplementary Figure S19. Uncropped western blot images of Suppl. Fig. S5 right panel showing ATXN7, GFAP and GAPDH expression in the striatum.
Note: one western blot membrane was cut in two horizontal pieces for three immunodetections.
